# Liver-resident cellular determinants of oncolytic virotherapy in hepatocellular carcinoma: viral susceptibility and spatial immune remodeling

**DOI:** 10.3389/fcell.2026.1959317

**Published:** 2026-10-07

**Authors:** Ding Hu, Qiong Wang

**Affiliations:** 1 Division of Liver Surgery, Department of General Surgery, West China Hospital, Sichuan University, Chengdu, China; 2 Department of Critical Care Medicine, West China Hospital, Sichuan University, Chengdu, China

**Keywords:** clinical translation, hepatic reserve, hepatocellular carcinoma, innate sensing, liver-resident cells, oncolytic virotherapy, spatial immune remodeling, viral susceptibility

## Abstract

Hepatocellular carcinoma (HCC) develops in a chronically injured and immunologically specialized organ that is both the treatment target and a major determinant of viral clearance and toxicity. Oncolytic viruses (OVs) can couple tumor-selective infection with immunogenic lysis, innate sensing, antigen presentation, and adaptive immune recruitment, but the clinical consequences of these processes depend on viral platform, delivery route, liver-resident cell handling, spatial disease distribution, and hepatic reserve. This review synthesizes HCC OV biology across four linked levels: platform-specific tumor-cell susceptibility; replication and interferon restriction; regulation by Kupffer cells, liver sinusoidal endothelial cells, dendritic-cell subsets, hepatic stellate cells, fibroblasts, and resident lymphoid populations; and spatial pharmacodynamics across injected tumors, non-injected intrahepatic lesions, cirrhotic parenchyma, and extrahepatic disease. We compare adenovirus, vaccinia virus, herpes simplex virus, and candidate RNA viruses using route- and assay-matched criteria and interpret clinical evidence according to study design, biological context, and permissible inference. Early Pexa-Vec studies established biological activity, whereas PHOCUS defined a negative boundary for Pexa-Vec followed by sorafenib in an unselected first-line population. H101 plus nivolumab and VG161 support early feasibility and pharmacodynamic investigation, while integrated human pharmacodynamic and diseased-liver HCC-model reovirus data highlight schedule-dependent immune effects and potential off-tumor liver inflammation. VIRAL-HCC is used only as a non-scored organizational matrix for prospective trial design and biomarker interpretation. Future development should link tumor exposure and productive infection or payload expression to compartment-resolved immune effects, clinically relevant tumor control, and preservation of hepatic reserve.

## 1 Introduction

Hepatocellular carcinoma (HCC) is treated within an organ that is simultaneously the site of malignancy, a central regulator of systemic metabolism and innate immunity, and a potential dose-limiting compartment. Contemporary treatment selection therefore integrates tumor burden and distribution with portal hypertension, hepatic reserve, performance status, transplant eligibility, and the feasibility of resection, ablation, transarterial therapy, or systemic treatment (D’Amico et al., 2018; Llovet et al., 2021a; Huang et al., 2022; Singal et al., 2023; European Association for the Study of the Liver, 2025; Reig et al., 2026). This tumor-organ duality distinguishes HCC from most extrahepatic solid tumors and makes preservation of non-tumoral liver function integral to the interpretation of any liver-directed investigational therapy.

Immune checkpoint inhibitor (ICI)-based regimens have improved outcomes in unresectable HCC, but efficacy is regimen-specific and durable benefit remains confined to a subset of patients (Finn et al., 2020; Yau et al., 2020; Abou-Alfa et al., 2022; Cheng et al., 2022; Kelley et al., 2022; Llovet et al., 2023; Qin et al., 2023; Gordan et al., 2024; Sangro et al., 2024; Yau et al., 2024; Qin et al., 2025; Yau et al., 2025). Resistance may arise from insufficient antigen presentation, Wnt/β-catenin-associated immune exclusion, exhausted T-cell states, suppressive myeloid programs, regulatory T cells, abnormal vasculature, and fibrotic stromal barriers (Spranger et al., 2015; Sia et al., 2017; Zheng et al., 2017; Kurebayashi et al., 2018; Ma et al., 2019; Ruiz de Galarreta et al., 2019; Zhang et al., 2019; Llovet et al., 2022; Xue et al., 2022; Zhu et al., 2022; Donne and Lujambio, 2023; Liu et al., 2023). These mechanisms are not interchangeable: a lesion can be poorly infiltrated because dendritic cells are absent, because T cells are trapped in stromal or perivascular niches, because myeloid cells suppress effector function, or because the surrounding diseased liver cannot tolerate productive inflammation. A strategy intended to sensitize HCC to immunotherapy must therefore be evaluated at the level of cell type, anatomical compartment, and time.

OVs offer a mechanistically attractive, but unproven, approach to this problem. Productive infection can cause tumor-cell lysis while releasing tumor antigens, pathogen-associated molecular patterns, and damage-associated molecular patterns that activate innate sensing and antigen presentation (Russell et al., 2012; Bommareddy et al., 2018; Gujar et al., 2018; Twumasi-Boateng et al., 2018). Early liver-cancer studies of JX-594/Pexa-Vec established infection, replication, transgene expression, tumor necrosis, and immune activation as biological proof-of-mechanism rather than definitive efficacy (Park et al., 2008; Heo et al., 2013). PHOCUS subsequently showed that sequential intratumoral Pexa-Vec followed by sorafenib did not improve outcomes in an unselected first-line population (Abou-Alfa et al., 2024). More recent H101, VG161, and reovirus studies broaden the translational questions to immune priming, payload expression, route, and dose scheduling, but none establishes comparative efficacy for an OV-containing regimen in HCC (Yi et al., 2024; Shen et al., 2025b; Scott et al., 2026).

This question requires a liver-centered framework. Tumor-cell susceptibility is conditioned by entry receptors, intracellular replication requirements, interferon competence, and engineering strategy. After administration, however, virions encounter complement, neutralizing antibodies, Kupffer cells, liver sinusoidal endothelial cells (LSECs), hepatic stellate cells, dendritic cells, resident lymphoid populations, and a cirrhosis-remodeled extracellular matrix. These compartments can restrict viral exposure, amplify or suppress innate sensing, and determine whether immune activation remains localized, reaches non-injected lesions, or precipitates hepatic injury. Accordingly, viral susceptibility, liver-resident cells, innate sensing, and spatial immune remodeling form the mechanistic center of this review; combinations and perioperative applications are treated as secondary, evidence-bounded development hypotheses.

### 1.1 What this review adds beyond previous reviews

Several recent HCC-focused reviews have summarized OV platforms, engineering strategies, delivery approaches, combination regimens, adverse events, and clinical development (Li et al., 2023; Zhu et al., 2023; Ye and Liu, 2025; Xiao et al., 2026; You et al., 2026). These reviews provide valuable platform-level and therapeutic overviews, but their organizing units are predominantly the virus, delivery technology, or treatment combination.

The present review uses a different organizing unit: the diseased liver as a spatially heterogeneous pharmacological and immunological system. We therefore integrate platform-specific tumor-cell susceptibility with Kupffer-cell and liver sinusoidal endothelial-cell handling, dendritic-cell cross-priming, fibrotic and fibroblastic barriers, resident lymphoid states, antiviral immunity, route-specific exposure, and hepatic reserve. We also distinguish injected or perfused tumor, non-injected intrahepatic lesions, non-tumoral cirrhotic parenchyma, and extrahepatic disease as separate pharmacodynamic compartments. Finally, key clinical studies are annotated independently by study design, biological context, and permissible inference, and VIRAL-HCC is used only to organize prospective trial hypotheses, biospecimens, and pharmacodynamic readouts. Table 1 summarizes how this emphasis differs from recent HCC-focused OV reviews.

**TABLE 1:** Scope of recent HCC-focused OV reviews and the incremental focus of the present review.

Review	Principal scope	Treatment of liver context	Incremental focus of the present review
Li et al., 2023	HCC OV status, intravenous delivery strategies, and emerging combinations	Emphasizes delivery barriers and systemic/IV strategies; liver biology is not organized primarily by resident cellular compartments	Integrates route-specific exposure with Kupffer cells, LSECs, dendritic cells, stroma, antiviral immunity, spatial disease compartments, and hepatic reserve
Zhu et al., 2023	Biological basis and oncolytic mechanisms, combination therapy, delivery, clinical trials, and challenges	Discusses HCC tumor biology, delivery, and immune mechanisms within a broad therapeutic overview	Reorganizes mechanism around cell-resolved liver handling and spatial pharmacodynamics across tumor and non-tumoral liver compartments
Ye and Liu, 2025	Common OV platforms, mechanisms, combination strategies, and adverse events in HCC.	Provides a broad HCC-oriented treatment overview without a dedicated compartment-resolved spatial framework	Separates tumor susceptibility, liver-resident clearance/priming, fibrotic barriers, and spatial outcomes as distinct translational questions
Xiao et al., 2026	OV-based immunotherapy, engineering, delivery, combinations, and translational bottlenecks with attention to cirrhotic HCC.	Explicitly emphasizes cirrhosis-related barriers and optimization strategies	Adds paired tumor/non-tumoral liver pharmacodynamics, four spatial disease compartments, and study-level annotation by design, context, and permissible inference
You et al., 2026	New-generation OV platforms, therapeutic status, combinations, and challenges in liver cancer/HCC.	Broad platform-centered synthesis of preclinical and clinical development	Centers the diseased liver as the pharmacological unit and links resident-cell biology to route, schedule, biospecimen design, and hepatic reserve
Present review	Viral susceptibility, liver-resident cellular control, spatial immune remodeling, route-specific pharmacodynamics, and post-PHOCUS trial annotation	The liver is treated simultaneously as target organ, clearance/immune compartment, spatial barrier, and dose-limiting tissue	Provides a liver-centered, cell- and compartment-resolved framework and separates study design, biological context, and permissible inference

## 2 Literature search and evidence appraisal

### 2.1 Search strategy and eligibility

This review was developed as a structured critical narrative synthesis rather than a systematic review or meta-analysis. For the revision audit, the prespecified database-specific searches were rerun on 5 September 2026 in PubMed/MEDLINE, Embase, and the Web of Science Core Collection. The searches identified 1,480 PubMed/MEDLINE records, 2,526 Embase records, and 2,106 Web of Science records (6,112 raw bibliographic records). After multistep deduplication using DOI, PMID, normalized title, first-author and publication-year information, followed by manual adjudication of ambiguous matches, 2,552 duplicate records were removed. The remaining 3,560 unique records underwent an audited title/abstract eligibility assessment; 2,942 were excluded and 618 candidate reports were retained for source-level eligibility and publication-lineage verification. Search concepts combined HCC or liver cancer with oncolytic virus, virotherapy, adenovirus, H101, dl1520/ONYX-015, vaccinia virus, JX-594/Pexa-Vec, herpes simplex virus, VG161, reovirus, vesicular stomatitis virus, Newcastle disease virus, measles virus, and coxsackievirus. Controlled vocabulary was used when available, and bibliographic database searches were restricted to English-language records. The complete search strings, interfaces, retrieval counts, registry queries, eligibility rules, source-level exclusion log, evidence annotation, and literature-selection flow diagram are provided in Supplementary Material 1.

Eligible evidence comprised peer-reviewed clinical studies of OVs in HCC, HCC-specific preclinical studies, clinically informative studies of liver-directed OV delivery in non-HCC liver tumors, and mechanistic studies that directly informed viral susceptibility, innate sensing, hepatic clearance, spatial immune remodeling, or liver-specific safety. Non-oncolytic viral gene therapy and therapeutic vaccination were excluded unless necessary to explain a platform-specific delivery or biosafety principle. Non-HCC studies were retained only when the extrapolated mechanism or delivery question was explicit. Conference abstracts and registry records were treated as complete available sources when no stand-alone full article existed and were used to describe development activity, protocol structure, safety, pharmacodynamics, or unresolved hypotheses rather than comparative efficacy. Detailed numerical, causal, or study-level clinical statements were checked against the primary full article or an authoritative registry record when available.

Initial record identification and eligibility assessment were performed by DH. Reference lists of key trials and reviews were screened manually, and retracted publications were excluded. Multiple reports from the same clinical program were linked by registry identifier, platform, study population, recruitment period, and publication lineage; corrections and publisher notices were reviewed together with the primary article. For key clinical studies, DH and QW independently verified study design, numerical outcomes, publication status, registry status, correction status, and major safety findings against primary reports and publicly accessible records; disagreements were resolved by discussion. Formal dual-independent title/abstract and source-level screening of all retrieved records was not performed. The source-level audit excluded 23 reports that were duplicate/overlapping, conference-only without sufficient distinct mature evidence, or insufficiently relevant cross-context sources, leaving 595 reports in the final evidence map: 65 HCC-clinical reports, 292 HCC-preclinical reports, 57 liver-directed cross-context reports, and 181 mechanistic cross-context reports. After conservative publication-lineage adjudication, the 65 HCC-clinical reports represented 26 unique clinical study/evidence units; the remaining nonclinical and cross-context sources were retained at report level, yielding 556 final evidence units. A literature-identification flow diagram is provided for audit transparency, but no standardized risk-of-bias instrument or quantitative pooling was applied; the review therefore remains a structured critical narrative synthesis rather than a systematic review.

### 2.2 Evidence annotation framework

To avoid conflating study design, disease context, and inferential strength, evidence was annotated across three independent dimensions rather than assigned a single composite hierarchy. First, study design was described as randomized clinical trial, prospective non-randomized or early-phase study, retrospective/observational study, case report/series, or preclinical experimental study. Second, biological context was identified as HCC, mixed primary liver cancer with an HCC subgroup, non-HCC liver-directed clinical evidence, other-cancer clinical evidence, or HCC-specific experimental models. Third, the permissible inference was specified as comparative efficacy, feasibility and safety, viral pharmacodynamics, immune pharmacodynamics, mechanistic plausibility, or hypothesis generation. These dimensions are reported separately in the evidence tables. This descriptive framework is intended to prevent evidence migration—for example, treating melanoma pharmacodynamics, retrospective H101-TACE associations, or non-cirrhotic liver-metastasis delivery studies as HCC comparative-efficacy evidence—without implying a validated certainty-of-benefit grading system. Platform-specific mechanistic features relevant to HCC are summarized in Table 2.

**TABLE 2:** Mechanistic comparison of OV platforms relevant to HCC.

Platform	Entry and replication	Innate sensing and payload	Liver pharmacology and route	Evidence boundary
Adenovirus (dl1520/ONYX-015; H101)	CAR/integrin-dependent or engineered entry; nuclear DNA replication; E1-region modifications condition replication	DNA-sensing and IFN restriction are cell dependent; moderate payload flexibility	Complement, anti-Ad immunity, and Kupffer-cell capture; intratumoral or protocol-defined arterial delivery is most measurable	Prospective early HCC feasibility; H101–TACE evidence is observational and does not isolate a viral effect (Habib et al., 2002; Lin et al., 2015; Yi et al., 2024)
Vaccinia (JX-594/Pexa-Vec)	Broad entry; cytoplasmic DNA replication; TK disruption links replication to nucleotide metabolism	Large payload capacity; GM-CSF expression demonstrated; poxviral immune antagonism modifies sensing	Intratumoral delivery has the deepest HCC experience; systemic exposure is limited by host clearance and immunity	Early biological proof-of-mechanism; randomized PHOCUS evidence is negative for Pexa-Vec followed by sorafenib in an unselected population (Heo et al., 2013; Abou-Alfa et al., 2024)
HSV-1 (VG161)	Nectin-1/HVEM-related entry; nuclear replication; attenuated neurovirulence genes support selectivity	Large multi-armed payload capacity; local cytokine and checkpoint-module expression requires direct potency and safety assessment	Most defensible for safely injectable lesions; multifocal coverage remains limited	Prospective phase I feasibility and pharmacodynamics; no comparative efficacy; exploratory model requires external validation (Shen et al., 2025b)
Reovirus and other RNA-virus platforms	Rapid cytoplasmic replication; platform-specific receptors; dependence on tumor antiviral defects	RIG-I/MDA5/TLR-related sensing; generally lower payload capacity and a potentially narrow replication–sensing window	Systemic and regional delivery are platform specific; schedule may influence off-tumor liver inflammation	Predominantly HCC-preclinical or cross-context evidence; reovirus schedule data inform mechanism, not HCC comparative efficacy (Scott et al., 2026)
Delivery-enhanced or armed constructs	Carrier cells, shielding, retargeting, hypoxia adaptation, or added immune payloads	Designed to modify exposure or local immune function; each modification requires mechanism-matched potency assays	May address clearance, multifocality, or local expression but increases manufacturing and biosafety complexity	Mostly preclinical or conceptual; VG161 is an early clinical example of multi-arming

## 3 Viral susceptibility and innate sensing in HCC

### 3.1 Entry and tumor-cell permissiveness

OV selectivity is conditional rather than absolute. Entry depends on platform-specific attachment and internalization pathways, including the coxsackievirus and adenovirus receptor and integrins for conventional adenoviruses, nectin-1 or HVEM for HSV, low-density lipoprotein receptor family members for vesicular stomatitis virus, and junctional adhesion molecule A for reovirus (Bergelson et al., 1997; Barton et al., 2001; Finkelshtein et al., 2013). Receptor abundance can affect infection, but it is not a sufficient biomarker because accessibility, internalization, intracellular trafficking, antiviral signaling, proliferative state, and local delivery also determine productive infection. In HCC, receptor expression may vary across tumor clones, dedifferentiated regions, vascular niches, and treatment-exposed lesions. Baseline receptor staining should therefore be considered a platform-specific enrichment variable, not evidence that a tumor will support replication.

Tumor-cell permissiveness also depends on the intracellular environment. Defects in viral sensing or downstream JAK–STAT signaling can permit viral genomes and proteins to accumulate before an antiviral state is established (Stojdl et al., 2003; Lichty et al., 2014). Oncogenic alterations involving Rb–E2F, RAS/MAPK, PI3K–AKT, p53-associated stress responses, and nucleotide metabolism may further alter replication, protein synthesis, and resistance to virus-induced death, but the relevant dependency varies by backbone (Alemany et al., 2000b; Kelly and Russell, 2007; Kirn et al., 2007; Russell et al., 2012; Kaufman et al., 2015; Fukuhara et al., 2016; Twumasi-Boateng et al., 2018). The same lesion may therefore be permissive to one platform and restrictive to another. A generic label of “OV-sensitive HCC” is therefore biologically inadequate. Susceptibility should be defined for a specific vector, route, dosing schedule, and intended payload and should be confirmed on treatment rather than inferred from a baseline receptor stain alone.

### 3.2 Replication compartment, engineering logic, and interferon restriction

Replication compartment shapes both selectivity and innate sensing. Adenovirus and HSV replicate predominantly in the nucleus, vaccinia replicates in the cytoplasm, and most candidate RNA viruses replicate in the cytoplasm. These differences determine which host enzymes are required, which pattern-recognition receptors encounter viral intermediates, how quickly interferon-stimulated genes restrict spread, and how readily transgenes can be inserted or expressed (Kelly and Russell, 2007; Russell et al., 2012; Kaufman et al., 2015; Fukuhara et al., 2016). Engineering strategies—including E1-region modification in adenovirus, thymidine kinase disruption in vaccinia, attenuation of HSV neurovirulence genes, tumor-selective promoters, and receptor retargeting—seek to widen the therapeutic index, but no modification abolishes dependence on host biology.

Type I interferon is a dual determinant rather than a unidirectional barrier. A weak tumor-cell antiviral program may permit replication and oncolysis, but insufficient sensing can reduce chemokine induction, dendritic-cell licensing, and adaptive immune priming. Conversely, a strong baseline interferon-stimulated gene program can restrict spread while supporting antigen presentation and effector recruitment once infection is established (Stojdl et al., 2003; Kato et al., 2006; Kawai and Akira, 2010; Diamond et al., 2011; Fuertes et al., 2011; Sun et al., 2013; Lichty et al., 2014; Woo et al., 2014; Bommareddy et al., 2018; Gujar et al., 2018; Rehwinkel and Gack, 2020). The relevant variable is therefore timing: productive infection must precede, or transiently coexist with, innate sensing sufficient to convert local damage into immune instruction. The proposed distinction between permissive-but-poorly-immunogenic and restrictive-but-immune-primable HCC states is an author hypothesis, not a validated clinical classification. It should be tested using paired measurements of viral replication, interferon-stimulated genes, antigen-presentation programs, and immune-cell recruitment.

### 3.3 Platform-specific sensing and viral immune antagonism

Innate sensing cannot be generalized across OV backbones. Adenovirus and HSV deliver DNA to the nucleus, but incoming capsids, endosomal escape, cytosolic DNA, and abortive replication can activate Toll-like receptors and cGAS–STING before or during nuclear replication. Vaccinia replicates in the cytoplasm, where DNA-sensing pathways are relevant but are countered by multiple poxviral immune-evasion proteins. Reovirus, vesicular stomatitis virus, and Newcastle disease virus generate cytoplasmic RNA species that engage RIG-I-, MDA5-, or Toll-like receptor-dependent pathways in a platform- and cell-dependent manner (Kato et al., 2006; Kawai and Akira, 2010; Sun et al., 2013; Rehwinkel and Gack, 2020). The net signal is determined not only by the host pathway but also by the viral antagonists retained or removed during engineering.

This distinction matters in HCC because the same sensing pathway may operate differently in malignant hepatocytes, Kupffer cells, dendritic cells, LSECs, and non-tumoral hepatocytes. A cytokine increase in blood cannot identify the producing compartment or establish tumor-directed immunity. Platform development should therefore pair viral kinetic assays with cell-resolved sensing readouts and should report whether inflammatory programs localize to infected tumor, peritumoral stroma, or cirrhotic parenchyma. Direct comparative human HCC data across platforms are not available; cross-platform rankings based on generic ‘immunogenicity’ are consequently unsupported.

### 3.4 Immunogenic lysis, payload expression, and adaptive priming

Viral lysis becomes immunologically relevant when tumor antigens are released with signals that recruit and activate antigen-presenting cells. Calreticulin exposure, extracellular ATP, HMGB1, viral nucleic acids, capsid proteins, and replication intermediates can engage endosomal Toll-like receptors, RIG-I-like receptors, cGAS–STING, NF-κB, IRF3/7, and inflammasome-associated programs (Kato et al., 2006; Apetoh et al., 2007; Obeid et al., 2007; Kawai and Akira, 2010; Diamond et al., 2011; Fuertes et al., 2011; Michaud et al., 2011; Sun et al., 2013; Woo et al., 2014; Rehwinkel and Gack, 2020). These signals may promote dendritic-cell maturation, cross-presentation, CXCL9/CXCL10 production, and recruitment of CD8-positive T cells and natural killer cells. However, detection of cytokines or circulating viral nucleic acid does not by itself demonstrate productive intratumoral replication, antigen cross-presentation, or tumor-specific T-cell priming.

Payload design can localize immune activity that would be difficult to deliver systemically in cirrhotic patients. Pexa-Vec encodes granulocyte–macrophage colony-stimulating factor, whereas VG161 encodes interleukin-12, interleukin-15, interleukin-15 receptor alpha, and a PD-1–PD-L1-blocking fusion protein (Trinchieri, 2003; Waldmann, 2006; Liu et al., 2008; Heo et al., 2011; Shen et al., 2023; Shen et al., 2025b). Large DNA-virus genomes provide greater transgene capacity than most RNA-virus platforms, but increasing payload complexity also creates demands for genomic stability, expression consistency, potency assays, and toxicity monitoring. Payload expression should therefore be measured directly in tumor tissue when feasible. Viral genome detection demonstrates exposure, not productive replication, infectious progeny, or functional transgene delivery.

The adaptive consequence of OV therapy should be described as potential immune remodeling, not assured conversion of a cold tumor into a hot tumor. Cross-tumor studies show that OV exposure can increase T-cell infiltration, interferon-related programs, and checkpoint expression, thereby creating a rationale for checkpoint blockade (Tumeh et al., 2014; Zamarin et al., 2014; Rooney et al., 2015; Ayers et al., 2017; Ribas et al., 2017; Yost et al., 2019; Chesney et al., 2023). In HCC, Pexa-Vec studies support infection and immune activation, but durable spatial immune conversion has not been established (Park et al., 2008; Heo et al., 2013). The strongest mechanistic evidence for T-cell clonal expansion and checkpoint sensitization derives from melanoma or other tumor models. HCC trials must therefore confirm antigen-presentation changes, T-cell localization, clonality, and myeloid remodeling directly rather than infer these effects from non-HCC experience.

Figure 1 summarizes this sequential but conditional mechanism, from local viral infection and immunogenic lysis to innate sensing, antigen presentation, adaptive immune remodeling, and compensatory checkpoint induction. It is intended as a mechanistic overview and does not imply that every step occurs with every viral platform or has been prospectively demonstrated in HCC.

**FIGURE 1:**
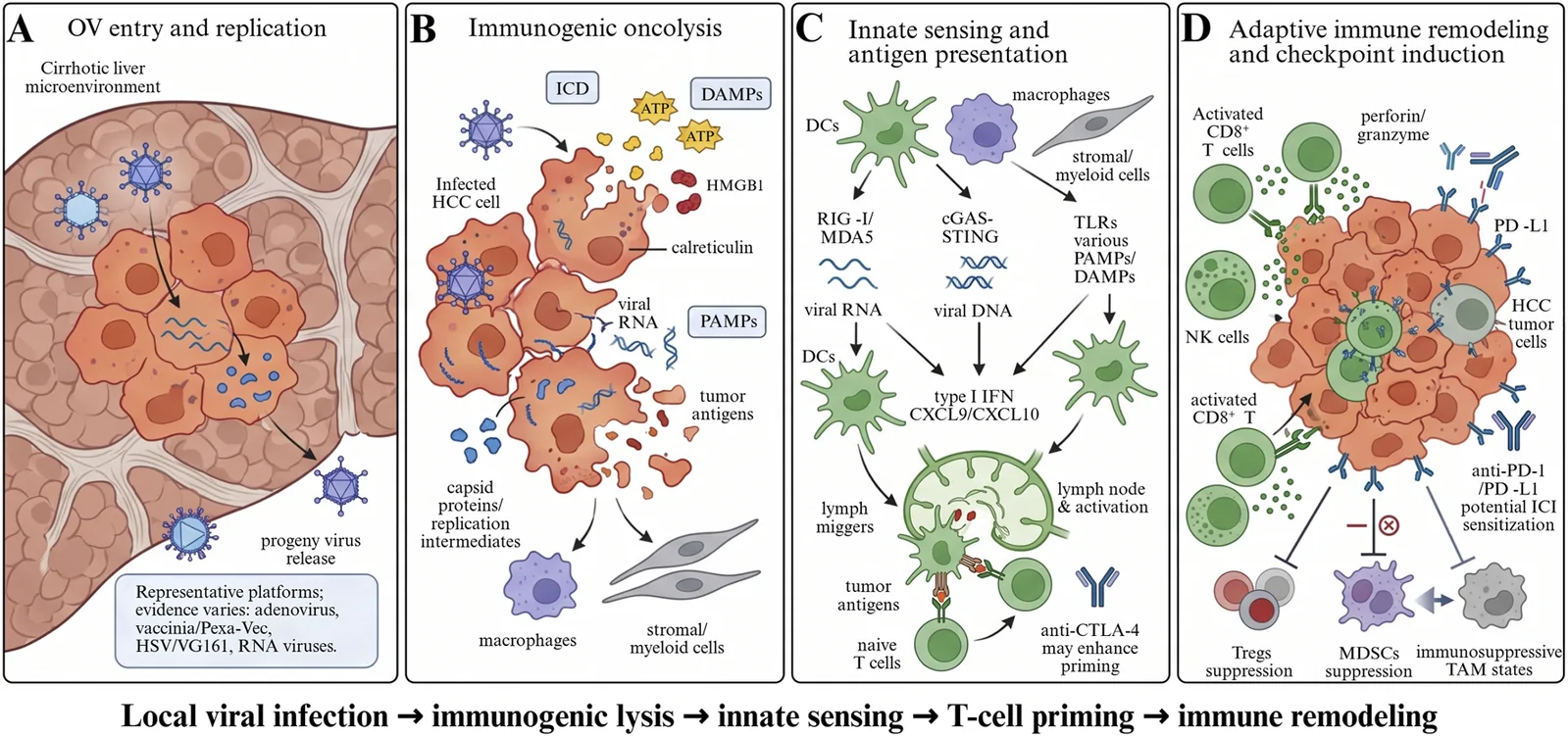
Sequential mechanisms of oncolytic-virus-induced immune remodeling in hepatocellular carcinoma. **(A)** Productive infection of permissive HCC cells may generate viral progeny and replication intermediates. **(B)** Immunogenic oncolysis releases tumor antigens together with viral pathogen-associated molecular patterns and damage-associated molecular patterns, including extracellular ATP, high-mobility group box 1, and surface calreticulin. **(C)** Viral RNA or DNA and tumor-derived danger signals engage dendritic, myeloid, and stromal sensing pathways, potentially promoting type I interferon signaling, antigen presentation, lymph-node priming, and recruitment of effector lymphocytes. **(D)** CD8-positive T cells and natural killer cells may contribute to tumor-cell killing, whereas regulatory T cells, myeloid-derived suppressor cells, immunosuppressive tumor-associated macrophage states, and adaptive checkpoint expression can constrain the response. The sequence is platform- and context-dependent, and the individual steps have not all been prospectively validated in HCC. Checkpoint-blockade annotations represent cross-context mechanistic rationales rather than direct evidence of additive efficacy in HCC. ATP, adenosine triphosphate; cGAS, cyclic GMP–AMP synthase; DAMP, damage-associated molecular pattern; DC, dendritic cell; HCC, hepatocellular carcinoma; HMGB1, high-mobility group box 1; ICI, immune checkpoint inhibitor; IFN, interferon; MDA5, melanoma differentiation-associated protein 5; MDSC, myeloid-derived suppressor cell; NK, natural killer; OV, oncolytic virus; PAMP, pathogen-associated molecular pattern; PD-1, programmed cell death protein 1; PD-L1, programmed death-ligand 1; RIG-I, retinoic acid-inducible gene I; STING, stimulator of interferon genes; TAM, tumor-associated macrophage; TLR, Toll-like receptor; Treg, regulatory T cell.

## 4 Liver-resident cellular control and spatial immune remodeling

### 4.1 Kupffer cells and tumor-associated macrophages

Kupffer cells are among the first cellular filters encountered by blood-borne virions. Adenovirus studies demonstrate rapid hepatic sequestration involving scavenger receptors, natural antibodies, complement, and Kupffer-cell uptake (Alemany et al., 2000a; Xu et al., 2008). This clearance protects the host but can reduce the fraction of administered virus reaching HCC deposits. Kupffer-cell depletion or indiscriminate suppression is not an immediately translatable solution because these cells also control infection, remove debris, and shape cytokine responses. The clinically relevant objective is to quantify when Kupffer-cell capture prevents tumor exposure and when it supplies innate signals that support antigen presentation.

Tumor-associated macrophages are distinct from resident Kupffer cells, although their phenotypes can overlap in diseased liver. HCC-associated macrophage populations can support angiogenesis, matrix remodeling, T-cell dysfunction, and resistance to immunotherapy (Kurebayashi et al., 2018; Ma et al., 2019; Zhang et al., 2019; Xue et al., 2022; Zhu et al., 2022; Donne and Lujambio, 2023; Liu et al., 2023). Viral infection may recruit inflammatory monocytes or alter macrophage state, but binary M1/M2 language does not capture the diversity resolved by single-cell and spatial studies. Future OV trials should distinguish resident Kupffer cells, recruited monocyte-derived macrophages, and intratumoral macrophage states using spatial markers and lineage-informed transcriptional programs; the conventional M1/M2 binary should not be used as the principal classification. A reduction in bulk macrophage abundance is not necessarily equivalent to productive immune remodeling.

### 4.2 Liver sinusoidal endothelial cells and dendritic-cell subsets

LSECs form a specialized vascular interface that scavenges circulating material, regulates leukocyte trafficking, and can present antigen in a tolerogenic context (Crispe, 2009; Heymann and Tacke, 2016; Sharma et al., 2020). In cirrhosis and HCC, sinusoidal capillarization, altered adhesion molecules, abnormal angiogenesis, and endothelial reprogramming change both viral access and immune-cell entry. LSECs may therefore act as a physical filter, an innate sensor, and a regulator of whether effector cells reach infected tumor regions. Arterial delivery cannot be assumed to bypass this biology, because viral distribution still depends on regional perfusion, endothelial permeability, shunting, and non-tumoral arterial exposure.

Dendritic-cell function should also be resolved by subset. Conventional type 1 dendritic cells are central to cross-presentation and type I interferon-dependent priming of CD8-positive T cells, whereas conventional type 2 and plasmacytoid dendritic cells contribute different antigen-presenting and interferon programs (Diamond et al., 2011; Fuertes et al., 2011; Woo et al., 2014). Their abundance and localization may be limited in immune-excluded HCC, particularly in Wnt/β-catenin-associated states (Pinyol et al., 2019; Ruiz de Galarreta et al., 2019). Viral lysis cannot generate systemic antitumor immunity if antigens are not captured and presented in an appropriate inflammatory context. Paired tissue studies should assess dendritic-cell subset, maturation state, proximity to infected tumor cells, and downstream T-cell clonotypes. Melanoma evidence on dendritic-cell exclusion provides cross-context mechanism, not direct evidence for HCC OV therapy (Spranger et al., 2015).

### 4.3 Hepatic stellate cells and cancer-associated fibroblasts

Hepatic stellate cells and cancer-associated fibroblasts shape the fibrotic and mechanical environment through collagen deposition, matrix cross-linking, growth factors, chemokines, and physical compartmentalization. Cirrhosis can increase interstitial pressure, alter sinusoidal flow, and create collagen-rich barriers between arterial vessels, tumor nests, and immune cells (Heymann and Tacke, 2016; Ringelhan et al., 2018; Sharma et al., 2020; Nguyen et al., 2021). These stromal effects may restrict viral dispersion after injection and trap lymphocytes in peritumoral or fibrous septal niches. The relevant endpoint is therefore not simply total viral DNA or total CD8-positive cell density, but the spatial relationship among infected tumor cells, fibroblastic barriers, antigen-presenting cells, and effector lymphocytes.

Stromal injury may also be double-edged. Viral or locoregional inflammation could transiently disrupt extracellular matrix and improve immune access, but it could also intensify wound-healing programs, hypoxia, angiogenesis, and fibrosis. Direct human HCC evidence that OVs remodel fibroblastic barriers is lacking. Claims that OVs normalize stroma or improve subsequent drug penetration should therefore be treated as preclinical or hypothesis-generating unless supported by paired human tissue.

### 4.4 Resident lymphoid cells, chronic liver disease, and etiology

Natural killer cells, natural killer T cells, tissue-resident memory T cells, exhausted virus-specific T cells, innate lymphoid cells, and regulatory T cells contribute to the hepatic immune baseline. These populations can support tumor cytotoxicity, but they may also clear infected cells before adequate viral spread or amplify injury in non-tumoral liver. Chronic HBV or HCV infection, alcohol-associated liver disease, and metabolic dysfunction-associated steatohepatitis produce distinct combinations of antigen exposure, innate activation, T-cell dysfunction, and myeloid remodeling (Heymann and Tacke, 2016; Ringelhan et al., 2018; Dudek et al., 2021; Heinrich et al., 2021; Pfister et al., 2021). In MASH-associated HCC, activated or PD-1-positive CD8-positive T cells may reflect pathogenic liver inflammation rather than effective tumor surveillance. Etiology should therefore be analyzed as a biological modifier of antiviral containment, immune priming, and liver toxicity—not merely as a demographic variable.

The same immune event can have different consequences in tumor and non-tumoral liver. Expansion of cytotoxic lymphocytes within infected tumor nests may support antitumor activity, whereas diffuse activation in cirrhotic parenchyma may precipitate hepatitis or decompensation. Blood immune profiling cannot resolve this distinction. Spatial transcriptomics, multiplex imaging, and paired tumor and non-tumoral liver sampling should be incorporated when clinically and ethically feasible, particularly in short-window preoperative studies. These approaches are research tools and should not delay curative treatment.

### 4.5 Spatial outcomes of local infection

A liver-directed OV produces at least four spatially distinct pharmacological compartments: the injected or perfused tumor region, non-injected intrahepatic lesions, surrounding cirrhotic parenchyma, and extrahepatic disease. High viral exposure in the first compartment does not establish coverage of the others. Intratumoral injection is well suited to proof-of-mechanism studies but may leave multifocal, infiltrative, bilobar, or extrahepatic disease underexposed. Hepatic arterial administration may broaden regional exposure, yet arterioportal shunts, prior embolization, vascular heterogeneity, necrosis, and portal vein tumor thrombus can create uneven deposition. Systemic administration offers broader theoretical coverage but faces complement, antibodies, and reticuloendothelial clearance (Alemany et al., 2000a; Xu et al., 2008; Ferguson et al., 2012; Llovet et al., 2021b).

Spatial immune remodeling should be defined by coordinated, compartment-specific changes rather than by one circulating or bulk-tissue marker. Evidence of productive remodeling would include platform-specific confirmation of infection or payload expression, activation of antigen-presenting cells adjacent to infected tumor, movement of CD8-positive T cells from vascular or stromal niches into tumor nests, expansion of tumor-reactive clonotypes, reprogramming of suppressive myeloid states, and concordant activity in non-injected lesions. The VG161 phase I study provides the strongest current human HCC example of paired molecular and spatial immune profiling, but its single-arm design and corrected exploratory analyses do not validate those readouts as efficacy surrogates (Shen et al., 2025b; Shen et al., 2025c). Spatial findings must also be interpreted against inflammation in non-tumoral liver and longitudinal liver-function trajectories. Tumor shrinkage accompanied by sustained bilirubin worsening, ascites, encephalopathy, or loss of subsequent treatment eligibility does not meet the proposed standard of liver-safe benefit.

Figure 2 places these mechanisms within the HCC liver by contrasting liver-specific barriers—limited hepatic reserve, hepatic scavenging and immune regulation, humoral and interferon-mediated restriction, suppressive immune states, fibrotic architecture, and procedure-related risk—with investigational opportunities created by arterialized tumors, intratumoral access, protocol-defined regional delivery, locoregional workflows, and paired pharmacodynamic sampling. The figure does not establish any delivery route or combination as standard care.

**FIGURE 2:**
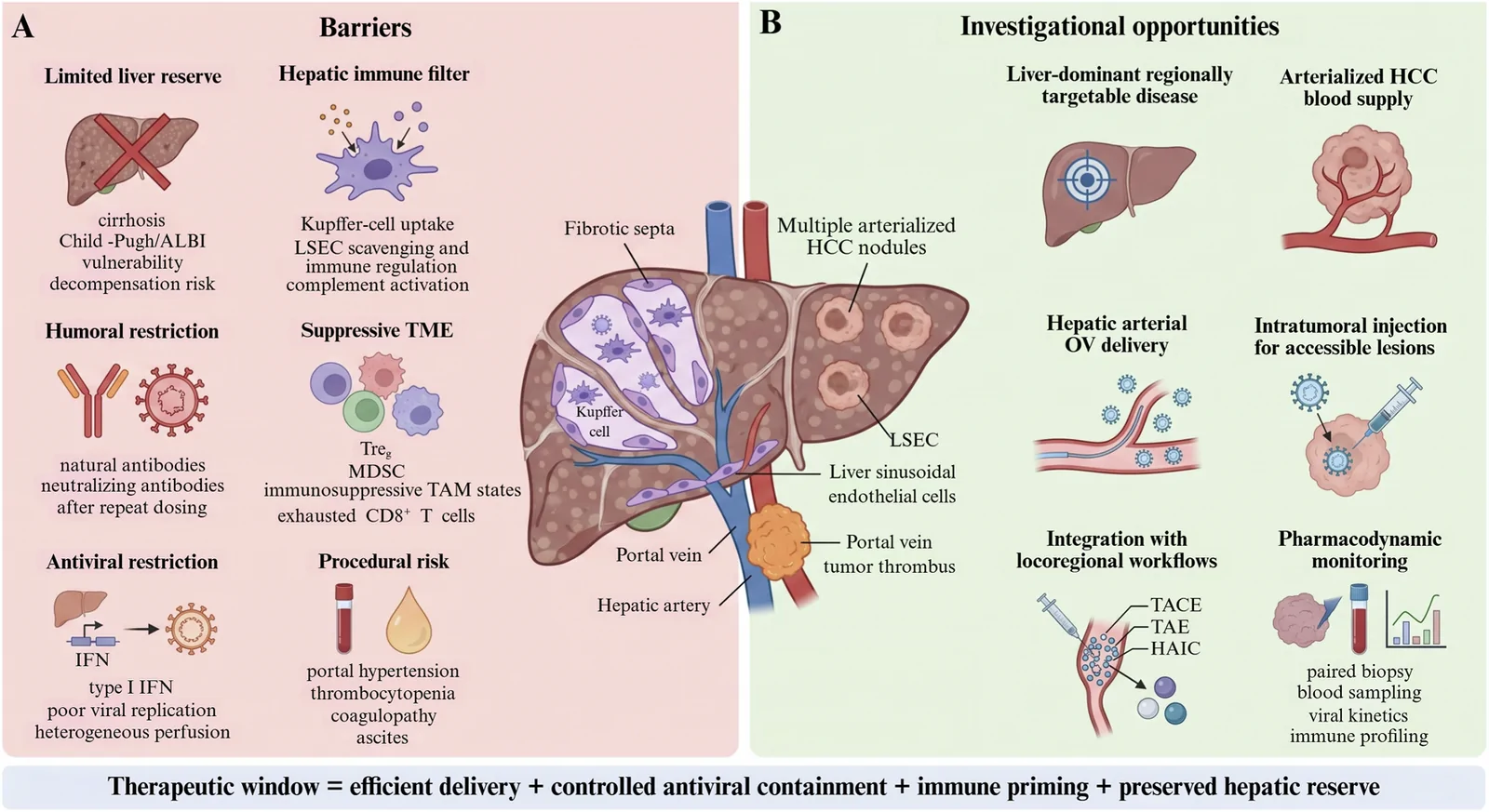
Liver-specific barriers and investigational opportunities for oncolytic virotherapy in hepatocellular carcinoma. **(A)** Limited hepatic reserve, cirrhosis-related procedural risk, complement- and antibody-mediated restriction, Kupffer-cell uptake, liver sinusoidal endothelial-cell scavenging and immune regulation, heterogeneous interferon-mediated restriction, suppressive immune states, and fibrotic architecture may reduce tumor exposure or amplify off-tumor liver injury. **(B)** Liver-dominant disease, arterialized tumor perfusion, intratumoral access, protocol-defined hepatic arterial administration, established locoregional workflows, and paired tissue and blood sampling provide investigational opportunities for route-specific pharmacodynamic studies. The routes and combinations shown are research strategies rather than established treatment recommendations. ALBI, albumin–bilirubin; HAIC, hepatic arterial infusion chemotherapy; HCC, hepatocellular carcinoma; ICI, immune checkpoint inhibitor; IFN, interferon; LSEC, liver sinusoidal endothelial cell; OV, oncolytic virus; TACE, transarterial chemoembolization; TAE, transarterial embolization; TKI, tyrosine kinase inhibitor; VEGF, vascular endothelial growth factor.

## 5 Mechanistic platform comparison and route-specific pharmacodynamics

### 5.1 Entry, replication compartment, and assay selection

Platform choice should begin with the biological problem to be solved. Adenoviruses offer mature engineering, high-titer manufacture, and compatibility with intratumoral or arterial delivery, but conventional serotype 5 vectors can be constrained by receptor heterogeneity, anti-adenoviral immunity, complement, and Kupffer-cell capture. HSV platforms provide substantial payload capacity and are suited to local multi-arming, but their most defensible current use requires safely injectable lesions. Vaccinia replicates in the cytoplasm, accommodates large transgenes, and has the deepest HCC clinical record; Pexa-Vec nevertheless demonstrates that biological activity does not guarantee efficacy. Candidate RNA viruses can replicate rapidly and generate strong pathogen-associated signals, but their HCC evidence is predominantly preclinical and their interferon sensitivity and systemic distribution require platform-specific validation (Habib et al., 2002; Ebert et al., 2003; Blechacz et al., 2006; Altomonte et al., 2010; Samson et al., 2018; Menotti and Avitabile, 2020; Andtbacka et al., 2021; Chen et al., 2022; Abou-Alfa et al., 2024; Shen et al., 2025b).

Replication compartment also affects the kinds of pharmacodynamic assays that are informative. Nuclear DNA-virus platforms can be assessed through intratumoral viral genome, transcript, protein, and payload expression, whereas cytoplasmic platforms require assays matched to their replication intermediates and infectious progeny. For all platforms, circulating viral nucleic acid is an exposure marker rather than proof of tumor replication. Infectious-virus recovery, viral protein or transcript, and functional payload expression provide stronger evidence when technically feasible. These measurements should be prespecified before dose expansion rather than added retrospectively after clinical outcomes are known.

### 5.2 Interferon state and payload logic

Platform behavior may differ according to baseline interferon state. A strongly interferon-restricted lesion may require local high-concentration delivery, altered scheduling, shielding, or vector engineering to achieve measurable exposure, whereas a poorly sensing lesion may permit replication yet generate insufficient dendritic-cell activation or type 1 immune signaling. These relationships are hypotheses for prospective stratification and pharmacodynamic testing rather than a validated patient-selection rule; no cross-platform interferon threshold or assay currently identifies an optimal OV for an individual HCC. Trials should therefore measure interferon-related programs together with platform-specific replication or payload readouts rather than collapse them into an unvalidated binary score.

Payload selection should address a defined bottleneck. GM-CSF is intended to recruit and activate antigen-presenting cells; IL-12 can promote type 1 immunity; IL-15/IL-15Rα can support natural killer and memory CD8-positive T-cell biology; and local checkpoint-blocking modules can limit adaptive resistance (Trinchieri, 2003; Waldmann, 2006; Liu et al., 2008; Heo et al., 2011; Shen et al., 2023; Shen et al., 2025b). More payloads do not necessarily produce better therapy. Each module adds manufacturing complexity, potential cytokine toxicity, and a requirement for direct expression and potency assays. In cirrhotic HCC, a defensible armed OV should have a measurable local pharmacodynamic purpose and a predeclared liver-safety boundary.

### 5.3 Hepatic clearance, route, and dosing schedule

Route is part of the mechanism rather than a logistical afterthought. Intratumoral delivery maximizes local concentration and supports paired biopsy, but it is lesion-limited. Hepatic arterial administration may increase regional exposure in arterialized tumors, but direct HCC evidence remains sparse and cannot be inferred from non-HCC liver metastasis studies (Reid et al., 2002; West et al., 2025). Transarterial co-administration with embolization is plausible, but the timing of viral infusion relative to vascular shutdown could increase or reduce distribution; this remains a hypothesis. Systemic administration provides the broadest theoretical coverage but is most vulnerable to complement, neutralizing antibodies, Kupffer-cell uptake, splenic sequestration, and dilution (Alemany et al., 2000a; Xu et al., 2008; Breitbach et al., 2011; Ferguson et al., 2012; Evgin et al., 2016).

Dose frequency is also part of the mechanism. A 2026 translational reovirus study found that repeated consecutive infusions did not increase the amplitude of systemic immune activation relative to a single dose and, in diseased-liver HCC models, were associated with greater activated T-cell influx and biochemical liver injury without improved antitumor efficacy (Scott et al., 2026). The patient immune analyses were not a randomized HCC efficacy comparison, and the liver-safety findings were largely preclinical; nevertheless, the study directly challenges the assumption that more viral doses necessarily provide greater immune benefit. Schedule selection should be justified by platform kinetics and supported by serial viral, immune, and liver-injury measurements.

The most defensible positioning is investigational and setting-specific. Adenovirus may be suited to measurable locoregional immune priming; vaccinia remains a reference platform for HCC pharmacodynamics and the PHOCUS boundary; HSV-1 supports localized multi-armed remodeling in accessible lesions; and RNA viruses remain candidates for biomarker-defined early translation after route, schedule, and liver safety are established. No platform can be ranked across HCC without considering lesion accessibility, disease distribution, antiviral immunity, payload purpose, dosing schedule, and hepatic reserve. Table 1 summarizes these mechanistic distinctions.

## 6 Clinical and translational evidence in HCC

### 6.1 Adenoviral studies: Feasibility and attribution

Early dl1520/ONYX-015 studies addressed whether replication-selective adenoviruses could be delivered to liver tumors. Intratumoral HCC experience and hepatic arterial studies dominated by gastrointestinal liver metastases demonstrated technical feasibility and enabled viral or immune measurements, but they were conducted before contemporary HCC therapy and lacked standardized paired tissue and spatial profiling (Habib et al., 2001; Habib et al., 2002; Reid et al., 2002). H101/rhAd5 was subsequently combined with transarterial chemoembolization (TACE) in case-based, retrospective, and non-randomized studies (He et al., 2011; Dong et al., 2014; Lin et al., 2015; He et al., 2017; Wu et al., 2021). Heterogeneity in TACE technique, embolic material, retreatment, liver reserve, and response assessment prevents isolation of the viral contribution. Collectively, these data support feasibility and the rationale for comparator-controlled locoregional studies that incorporate direct viral and immune pharmacodynamics.

### 6.2 Pexa-Vec and the PHOCUS boundary

JX-594/Pexa-Vec provides the most mature HCC-specific OV pharmacodynamic record. Early studies documented intratumoral infection, replication, GM-CSF expression, inflammatory and vascular effects, tumor necrosis, and activity in selected non-injected lesions (Liu et al., 2008; Park et al., 2008; Heo et al., 2011; Breitbach et al., 2013; Heo et al., 2013). The randomized dose-finding study supported continued development but did not compare Pexa-Vec with an active standard of care, and TRAVERSE did not demonstrate a definitive survival advantage after sorafenib failure (Heo et al., 2013; Moehler et al., 2019).

PHOCUS randomized 459 systemic-therapy-naive patients to intratumoral Pexa-Vec followed by sorafenib or sorafenib alone. Median overall survival was 12.7 versus 14.0 months (hazard ratio 1.193, 95% confidence interval 0.928–1.535), time to progression was 2.0 versus 4.2 months, and no improvement was demonstrated in objective response or disease control; serious adverse events occurred in 53.7% and 35.5% of patients, respectively, with liver failure the most frequently reported serious event in both groups (Abou-Alfa et al., 2024). The trial therefore defines a negative boundary for this specific sequence in an unselected first-line population. Proposed explanations—including incomplete lesion coverage, antiviral clearance, absence of biomarker enrichment, insufficient immune remodeling, or partner selection—remain mechanistic hypotheses because they were not prospectively adjudicated.

### 6.3 H101 plus nivolumab and VG161

The H101 plus nivolumab pilot study (registered as ChiCTR1900025377) evaluated intratumoral adenoviral priming followed by repeated H101 and nivolumab in refractory advanced HCC. Among 18 assessable patients, the objective response rate was 11.1%, disease control rate 38.9%, median progression-free survival 2.69 months, and median overall survival 15.04 months; low-grade fever and puncture-related pain were common, and no grade 3 or 4 adverse events were reported in the small cohort (Yi et al., 2024). As a single-arm study, its strongest contribution is procedural feasibility and an exploratory immune-priming rationale rather than estimation of the incremental effect of H101.

VG161 represents a different development strategy: a multi-armed HSV-1 encoding IL-12, IL-15, IL-15Rα, and a PD-1-PD-L1-blocking fusion protein. A multicenter phase I study in treatment-refractory primary liver cancer, including an HCC cohort, reported tolerability, radiographic activity, and immune pharmacodynamic findings; interpretation should use the corrected publication (Shen et al., 2025b; Shen et al., 2025c). The study is an important example of tumor-based pharmacodynamic and spatial immune profiling. Its gene-expression model, however, was developed within the same early-phase program and remains exploratory pending prespecified thresholds, external validation, and evidence of clinical utility.

A reported VG161 plus camrelizumab study is available at conference-abstract level, and relevant trial-registry records are used here to describe development activity rather than treatment effect (Shen et al., 2025a; ClinicalTrials.gov, 2026a; ClinicalTrials.gov, 2026b).

The revision search also identified additional HCC clinical evidence that sharpens these boundaries. In a prospective randomized phase III trial of 162 patients with solitary HCC no larger than 3 cm, H101 combined with radiofrequency ablation did not significantly improve 3-year disease-free survival (54.0% versus 41.2%, P = 0.090) or overall survival (93.0% versus 92.9%, P = 0.688) compared with radiofrequency ablation alone (Dai et al., 2026). A separate single-arm study of intravenous M1-c6v1 combined with camrelizumab and apatinib enrolled 13 patients with advanced HCC and reported an objective response rate of 70% among 10 evaluable patients, with median progression-free and overall survival of 8.9 and 15.4 months, respectively; the uncontrolled design permits feasibility, safety, and preliminary-activity inference but not attribution of benefit to the viral component (Xie et al., 2025). MASTERKEY-318 evaluated intrahepatic T-VEC alone and with pembrolizumab in mixed liver-tumor cohorts; the HCC combination cohort showed limited non-comparative activity, supporting route-specific feasibility but not additive efficacy (Hecht et al., 2025).

### 6.4 Reovirus schedule evidence and the boundary between human pharmacodynamics and HCC efficacy

The reovirus scheduling study by Scott et al. integrated immune analyses from single- and repeated-dose clinical cohorts with mechanistic experiments in HCC models of steatotic or fibrotic liver (Scott et al., 2026). Single infusion was equivalent or superior to repeated consecutive dosing for several inflammatory and circulating immune readouts; in diseased-liver HCC models, repeated dosing did not improve antitumor effects and increased activated T-cell accumulation and liver injury. The human and experimental components therefore inform scheduling and liver-safety hypotheses, but they do not establish an optimal clinical dosing schedule for HCC.

### 6.5 Clinical synthesis

Taken together, current HCC data establish that local administration of several OV platforms is technically feasible and can generate measurable viral or immune pharmacodynamics; PHOCUS shows that biological activity does not guarantee clinical benefit; and route and schedule materially influence exposure and liver inflammation. The next clinical questions are whether clinically relevant lesions are reached, whether productive infection or functional payload expression is demonstrated in tumor, whether immune changes are spatially tumor-directed, and whether these effects occur without clinically meaningful deterioration in hepatic reserve. Table 3 summarizes the study design, biological context, and permissible inference for the principal clinical and translational studies.

**TABLE 3:** Peer-reviewed clinical and translational evidence relevant to OV development in HCC. Study design, biological context, and permissible inference are annotated separately; clinical publication lineage and registry status are detailed in Supplementary Tables S4 and S5, and non-HCC liver-directed/cross-context evidence is summarized in Supplementary Table S6.

Study/evidence	Platform and route	Design	Key observation	Permitted inference	Biological context
dl1520 intratumoral HCC studies (Habib et al., 2001)	Adenovirus; intratumoral	Early-phase HCC	Feasible delivery and preliminary biological assessment	Feasibility and preliminary biological assessment	HCC clinical
JX-594 phase I and randomized dose-finding (Park et al., 2008; Heo et al., 2013)	Vaccinia; intratumoral	Early phase and randomized dose-finding	Replication, GM-CSF expression, vascular effects, immune activation, and dose-related signals	Proof of infection, replication, payload expression, and dose-related biological activity; active-comparator efficacy was not tested	HCC/liver-cancer clinical
TRAVERSE (Moehler et al., 2019)	Vaccinia; intratumoral	Randomized phase IIb after sorafenib failure	No definitive survival advantage over best supportive care	Survival benefit was not demonstrated in the tested post-sorafenib setting	HCC clinical
PHOCUS (Abou-Alfa et al., 2024)	Vaccinia; Pexa-Vec followed by sorafenib	Randomized phase III; n = 459	No OS, TTP, ORR, or DCR improvement; more serious adverse events; trial stopped early	The tested Pexa-Vec-to-sorafenib sequence did not improve outcomes; mechanisms of failure remain unresolved	HCC clinical
H101/rhAd5 plus TACE reports (He et al., 2011; Dong et al., 2014; Lin et al., 2015; Wu et al., 2021)	Adenovirus; transarterial with TACE	Retrospective, non-randomized, prognostic, or case-based	Feasibility and outcome associations in heterogeneous cohorts	Feasibility and outcome associations; the independent viral contribution cannot be isolated	HCC clinical
H101 plus nivolumab (Yi et al., 2024)	Adenovirus; intratumoral plus PD-1 blockade	Single-arm pilot; 18 assessable patients	Feasible; ORR 11.1%, DCR 38.9%, median PFS 2.69 months, median OS 15.04 months	Feasibility and exploratory immune-priming rationale; the incremental contribution of H101 cannot be estimated from the single-arm design	HCC clinical
VG161 phase I (Shen et al., 2025b)	Multi-armed HSV-1; intratumoral	Multicenter non-randomized phase I	Tolerability, radiographic activity, and immune/spatial pharmacodynamic findings	Feasibility and exploratory pharmacodynamic/biomarker findings requiring external validation	Primary liver cancer with HCC cohort
Single versus repeated reovirus dosing (Scott et al., 2026)	Reovirus; intravenous clinical cohorts plus HCC models	Integrated human pharmacodynamic and HCC-preclinical study	Repeated dosing did not enhance immune amplitude and increased background-liver immune injury in HCC models	Scheduling and liver-safety hypotheses; no HCC comparative-efficacy inference	Non-HCC clinical pharmacodynamics + HCC preclinical models
H101 + RFA randomized phase III (Dai et al., 2026)	Adenovirus; local H101 combined with RFA	Prospective randomized phase III; n = 162	No statistically significant 3-year DFS or OS improvement versus RFA alone	Comparative evidence for the tested H101+RFA regimen; benefit was not demonstrated in the reported analysis	HCC clinical
T-VEC ± pembrolizumab, MASTERKEY-318 (Hecht et al., 2025)	HSV-1; intrahepatic/intratumoral ± systemic PD-1 blockade	Phase Ib/II multicenter open-label basket trial	Limited non-comparative activity in the HCC cohort; route-specific DLTs and safety were characterized	Route-specific safety/feasibility and cohort activity; additive efficacy cannot be estimated	Mixed liver-tumor clinical evidence with HCC cohort
M1-c6v1 + camrelizumab + apatinib (Xie et al., 2025)	Alphavirus M1-c6v1; intravenous + PD-1/VEGFR2 blockade	Single-arm investigator-initiated clinical trial; n = 13	Preliminary activity and tolerability were reported; 10 patients were efficacy-evaluable	Feasibility, safety, pharmacodynamics and preliminary activity; no comparative or component-attribution inference	HCC clinical

## 7 Organizational framework for post-PHOCUS trial design

### 7.1 VIRAL-HCC as a non-scored trial-annotation matrix

VIRAL-HCC is used here as a six-domain organizational matrix comprising viral permissiveness, interferon state, regional delivery feasibility, antiviral immunity, liver reserve, and HCC immune phenotype. It is deliberately non-scored and has no weights, thresholds, cutoffs, calibration, discrimination estimates, or external validation. Its intended use is to structure hypothesis definition, biospecimen timing, pharmacodynamic readouts, and failure analysis within prospective trials; it is not intended to rank patients, viral platforms, or treatment options.

Baseline annotation can include platform-specific entry factors and tumor antiviral-pathway features, with receptor expression treated as one component rather than a stand-alone marker of productive infection. Interferon state can be characterized in tumor and microenvironmental compartments; regional delivery feasibility can capture lesion accessibility, arterial anatomy, arterioportal shunts, portal vein tumor thrombus, prior embolization, intrahepatic distribution, and extrahepatic disease; antiviral immunity can include serology or neutralization assays when platform relevant; liver reserve can include Child-Pugh class, ALBI grade, bilirubin, albumin, INR, platelets, portal-hypertension features, ascites, and encephalopathy; and the immune phenotype can incorporate intratumoral CD8-positive T-cell localization, dendritic-cell abundance, myeloid states, fibroblastic barriers, and Wnt/β-catenin-associated exclusion (Kamath et al., 2001; Johnson et al., 2015; Sia et al., 2017; Zheng et al., 2017; Kurebayashi et al., 2018; Harding et al., 2019; Ma et al., 2019; Pinyol et al., 2019; Ruiz de Galarreta et al., 2019; Zhang et al., 2019; Sharma et al., 2020; Nguyen et al., 2021; Llovet et al., 2022; Xue et al., 2022; Zhu et al., 2022; Donne and Lujambio, 2023; Liu et al., 2023).

On-treatment assessment can determine whether the prespecified biological chain occurred. Tumor exposure can be measured at lesion level or in tissue; productive infection can be supported by platform-matched replication-associated transcripts or proteins, replication intermediates, or infectious progeny when technically feasible; and functional payload delivery can be assessed by direct measurement of the encoded product and a mechanism-linked potency readout. Antiviral containment can be characterized separately through neutralizing antibodies, complement products, circulating virus, interferon-stimulated genes, and repeat-dose kinetics. Paired biopsies, multiplex imaging, spatial transcriptomics or single-cell approaches, T-cell receptor clonality, dendritic-cell activation, CD8-positive T-cell localization, and macrophage or fibroblast remodeling can then be used to localize immune effects. Sampling schedules should follow platform-specific replication and immune kinetics rather than a common template across backbones.

Endpoint interpretation can integrate mechanism, lesion-level tumor control, and hepatic reserve. Advanced-disease studies can distinguish injected from non-injected lesions and intrahepatic from extrahepatic disease and report RECIST 1.1, modified RECIST, durable response, progression-free survival, and overall survival as appropriate (Eisenhauer et al., 2009; Lencioni and Llovet, 2010; Seymour et al., 2017). Locoregional studies can prespecify viable-tumor response and time to untreatable progression. Short-window perioperative studies can use resection tissue to test mechanism while also reporting treatment-to-surgery interval, cancellation, R0 resection, 30- and 90-day morbidity and mortality, posthepatectomy liver failure, bile leakage, and viral shedding (Koch et al., 2011; Rahbari et al., 2011; Llovet et al., 2021c; Kaseb et al., 2022). Circulating tumor DNA and molecular residual disease remain exploratory endpoints in this setting (Cai et al., 2019; Ren et al., 2024; Hu et al., 2025). Table 4 maps the six domains across baseline, on-treatment, and endpoint assessment.

**TABLE 4:** VIRAL-HCC organizational domains across baseline, on-treatment, and endpoint assessment.

Domain	Baseline measures	On-treatment confirmation	Endpoint interpretation	Interpretive boundary
Viral permissiveness	Platform-specific entry factors; antiviral-pathway integrity; lesion heterogeneity	Tumor exposure: intratumoral viral genome. Productive infection: replication-associated transcript or protein, replication intermediates, or infectious progeny when feasible. Functional payload: direct expression and/or mechanism-linked potency	Were tumor exposure, productive infection, and/or functional payload expression demonstrated as prespecified for the platform?	Platform-specific; no validated cross-platform threshold
Interferon state	Tumor and microenvironmental ISG programs; JAK-STAT and sensing features	Temporal ISG, cytokine, and chemokine changes interpreted alongside replication kinetics and cellular compartment	Did sensing support immune priming, terminate replication prematurely, or produce off-tumor inflammation?	Nonlinear and platform specific; exploratory annotation
Regional delivery feasibility	Injectable lesions; arterial anatomy; shunts; PVTT; prior embolization; intrahepatic distribution; extrahepatic burden	Lesion-level exposure, injected versus non-injected lesion assessment, and route-specific biodistribution	Were the clinically relevant disease compartments reached?	Requires route-specific prospective confirmation
Antiviral immunity	Neutralizing antibodies or serology when relevant; complement susceptibility; prior viral exposure	Antibody kinetics, complement products, circulating virus as a systemic-exposure marker, viral clearance, and repeat-dose effects	Did antiviral containment prevent tumor exposure, limit repeat dosing, or contribute to immune licensing?	Assay interpretation is platform and schedule specific
Liver reserve	Child-Pugh class; ALBI grade; bilirubin; albumin; INR; platelets; portal hypertension; ascites; encephalopathy	Longitudinal liver tests, decompensation events, liver-specific dose-limiting toxicities, and stopping rules	Was tumor control achieved without decompensation or loss of subsequent treatment opportunity?	Clinical outcome domain; component definitions and adjudication must be prespecified
HCC immune phenotype	CD8-positive T-cell localization; dendritic-cell subsets; macrophage states; CAF/stromal barriers; Wnt/beta-catenin-associated exclusion	Spatial immune profiling, TCR clonality, antigen-presentation changes, and myeloid or stromal remodeling in tumor and non-tumoral liver	Did infection produce tumor-directed spatial immune remodeling without diffuse injurious liver inflammation?	Exploratory biomarker domain; no validated predictive model

The matrix is intended to make mechanistic assumptions explicit and testable. For example, baseline receptor expression is more interpretable when paired with direct evidence of intratumoral exposure or replication; immune activation is more informative when localized to antigen-presenting and effector-cell interactions in tumor than when inferred from blood cytokines alone; and immune activation in non-tumoral liver should be interpreted alongside longitudinal liver function. These propositions require prospective, platform-specific validation.

### 7.2 Hepatic reserve and liver-specific safety as parallel outcomes

Child-Pugh and ALBI are more informative when followed longitudinally rather than treated only as eligibility variables. In this review, “liver-safe benefit” is used only as shorthand—not as a validated composite endpoint—for interpreting tumor control alongside new or worsening ascites, encephalopathy, variceal bleeding, sustained bilirubin or INR deterioration, clinically meaningful ALBI or Child-Pugh worsening, hospitalization for decompensation, or loss of access to subsequent therapy. Protocols that use such an integrated assessment need prespecified component definitions, assessment intervals, competing-cause rules, and adjudication. Transient aminotransferase elevation should be interpreted separately from hepatic decompensation because viral inflammatory injury, tumor necrosis, immune-mediated hepatitis, ischemic injury, biliary obstruction, tumor progression, and cirrhosis-related deterioration have different implications.

Parallel assessment of tumor and liver outcomes is particularly important in combination studies because anti-VEGF therapy, TACE or TAE, radiotherapy, ICI, and surgery each introduce distinct liver- or procedure-related risks. Radiographic response should therefore be reported alongside liver-function trajectories, decompensation events, treatment interruption, and access to subsequent therapy. When attribution is uncertain, multidisciplinary adjudication can help distinguish treatment-related injury from tumor progression or underlying liver disease.

### 7.3 Trial conduct, manufacturing, and biosafety

For studies intended to estimate additive efficacy, contemporary matched comparators provide the most interpretable design. An OV-ICI trial can compare the combination with the same ICI-based backbone, and an OV-TACE study can use a protocol-defined TACE control; single-arm expansion remains appropriate when the primary objective is mechanism, dose, safety, or feasibility. Early-phase programs can prespecify evidence of tumor exposure, productive infection, or functional payload expression before large efficacy expansions and define stopping or redesign criteria for failure of delivery, absent pharmacodynamic activity, excessive antiviral clearance, or clinically meaningful loss of hepatic reserve.

Chemistry, manufacturing, and controls remain relevant to biological interpretation because genomic titer, infectious titer, genome and transgene stability, and mechanism-matched potency determine whether a nominal dose can produce the intended intratumoral effect (US Food and Drug Administration, 2020; International Council for Harmonisation, 2023). For liver-directed administration, product activity should be confirmed after clinically relevant handling, including thawing, catheter loading, contrast exposure, intratumoral injection, or arterial infusion. Multi-armed vectors require potency assays that demonstrate functional payload expression rather than replication alone.

Biosafety assessment should distinguish input virus, systemic exposure, productive infection, and viral shedding, and should define platform-specific monitoring after local, arterial, systemic, or repeated administration (US Food and Drug Administration, 2015). Chronic HBV status and antiviral treatment should be documented prospectively. Liver-transplant candidates constitute a separate high-risk population because replication-competent virus followed by immunosuppression creates unresolved clearance and rescue questions; current evidence does not support routine peri-transplant OV exposure.

## 8 Discussion

### 8.1 Integrated evidence synthesis

The current HCC OV literature supports a narrower set of conclusions than its mechanistic breadth might suggest. Selected platforms can infect liver tumors, express payloads, and induce measurable inflammatory or immune pharmacodynamics, whereas PHOCUS showed no outcome improvement for one Pexa-Vec-to-sorafenib sequence. Evidence that local infection reproducibly remodels non-injected disease or that adding an OV improves a contemporary ICI or locoregional backbone remains insufficient. This gap between biological activity and clinical benefit motivates a liver-centered evaluation that links exposure, productive infection or payload expression, spatial immune effects, and longitudinal hepatic reserve within the same development program.

In this framework, viral pharmacology is distributed across interacting cell compartments. Tumor cells determine entry and intracellular permissiveness; Kupffer cells and recruited macrophages regulate capture and inflammatory amplification; LSECs influence filtering and immune-cell trafficking; dendritic cells regulate cross-priming; stellate cells and cancer-associated fibroblasts shape mechanical and spatial barriers; and lymphoid populations determine whether inflammation remains tumor-directed or extends into background liver. The key translational unit is therefore a spatially organized tumor-liver system rather than cytolysis alone.

### 8.2 Four unresolved biological trade-offs

First, replication and immune sensing are mutually enabling but potentially antagonistic. Weak antiviral signaling can permit viral amplification yet fail to license antigen presentation, whereas early strong interferon responses can support cross-priming but terminate spread. Second, local exposure and systemic disease coverage are not equivalent: intratumoral injection enables high concentration and tissue sampling but may leave multifocal or extrahepatic disease untreated. Third, antiviral immunity can provide adjuvant signals while also neutralizing repeat doses. Fourth, the desired tumor-directed inflammatory response can become clinically harmful when it extends into cirrhotic parenchyma. These trade-offs cannot be resolved by selecting the most cytolytic backbone; they require platform-, route-, time-, and compartment-specific measurements.

The reovirus scheduling study illustrates the safety side of these trade-offs, whereas VG161 illustrates the value of direct tumor pharmacodynamics and spatial immune measurement (Shen et al., 2025b; Shen et al., 2025c; Scott et al., 2026). These studies are best interpreted as complementary mechanistic constraints: one questions whether repeated exposure always increases useful immunity, and the other shows how tissue-based readouts can test whether a multi-armed vector engages its intended biology.

### 8.3 Combination strategies: evidence-proximal and exploratory opportunities

OV-ICI combinations have the clearest mechanistic rationale because viral antigen release and innate sensing may generate or amplify an immune response that is subsequently constrained by checkpoint signaling. H101 plus nivolumab supports feasibility in HCC, while OV-ICI studies in melanoma and other tumors provide cross-context mechanistic support. Demonstration of incremental benefit in HCC, however, will require a comparator matched for the non-OV backbone together with direct evidence of tumor exposure or payload expression and, where feasible, paired spatial immune assessment.

Anti-VEGF or tyrosine kinase inhibition and locoregional procedures may alter perfusion, endothelial function, immune-cell trafficking, myeloid programs, antigen release, and regional viral access, but direct HCC OV evidence remains limited. Ablation, radiotherapy, conversion, perioperative, and molecular-residual-disease-directed approaches are even more exploratory. Their immediate value is therefore as mechanism-oriented research settings with prespecified safety safeguards and tissue or blood sampling, rather than as treatment algorithms.

Figure 3 summarizes these investigational settings as a trial-design map. It separates clinical setting and delivery route from the biological question being tested and does not assign a preferred treatment sequence.

**FIGURE 3:**
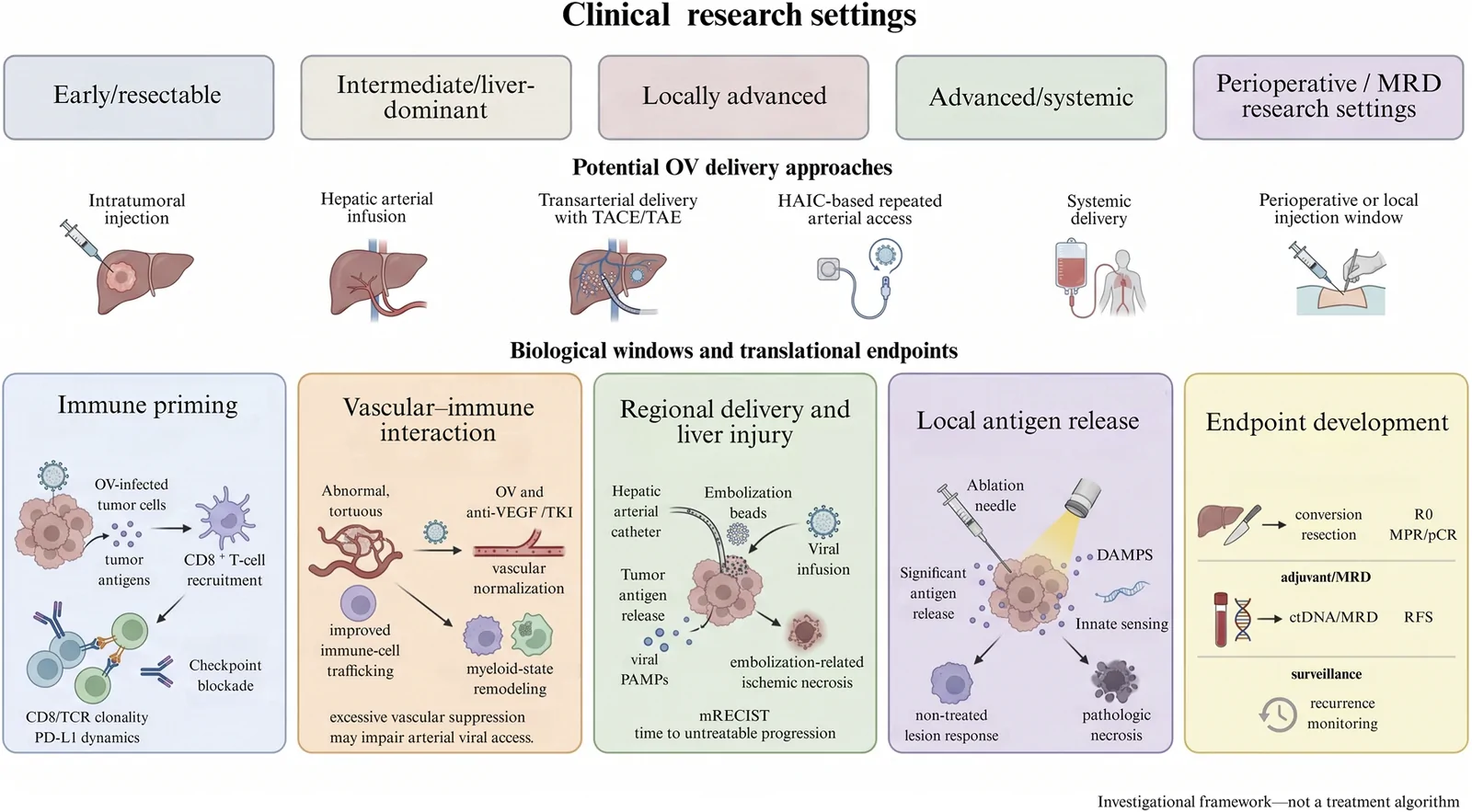
Mechanism-matched investigational oncolytic-virus strategies across hepatocellular carcinoma clinical settings. The figure maps selected clinical settings to potential delivery routes and to five translational questions: immune priming with checkpoint blockade, vascular-immune modulation, liver-directed delivery with locoregional procedures or repeated arterial access, local antigen release, and endpoint development in conversion, perioperative, adjuvant, or molecular-residual-disease studies. Evidence qualifiers are summarized in Table 3 rather than used as treatment recommendations. The figure is a trial-design framework and does not assign a preferred treatment sequence. ctDNA and MRD measures remain exploratory in HCC OV studies. cGAS, cyclic GMP-AMP synthase; CTLA-4, cytotoxic T-lymphocyte-associated protein 4; ctDNA, circulating tumor DNA; DAMP, damage-associated molecular pattern; HAIC, hepatic arterial infusion chemotherapy; HCC, hepatocellular carcinoma; ICI, immune checkpoint inhibitor; MPR, major pathological response; MRD, molecular residual disease; mRECIST, modified Response Evaluation Criteria in Solid Tumors; OV, oncolytic virus; PAMP, pathogen-associated molecular pattern; pCR, pathological complete response; PD-1, programmed cell death protein 1; PD-L1, programmed death-ligand 1; RFS, recurrence-free survival; STING, stimulator of interferon genes; TACE, transarterial chemoembolization; TAE, transarterial embolization; TCR, T-cell receptor; TKI, tyrosine kinase inhibitor; VEGF, vascular endothelial growth factor.

### 8.4 Limitations and priorities for the field

The clinical literature is small and heterogeneous with respect to backbone, engineering, route, dose, schedule, liver function, etiology, prior therapy, lesion accessibility, and response criteria. Only one large phase III trial is available, and it was negative for a defined sequence. Early studies often lack mandatory tumor pharmacodynamics and are vulnerable to attribution bias. Several proposed mechanisms derive from non-HCC OV studies or non-OV HCC studies. Platform-specific biology therefore precludes class-wide efficacy statements.

This review also has methodological limitations. Bibliographic searches were restricted to English-language records, which may underrepresent Chinese-language H101 and locoregional studies and can introduce language bias. Initial literature identification and eligibility assessment were performed by one author, while dual independent verification was limited to key clinical studies; formal dual-independent screening of all retrieved records was not performed. The revision audit provides database-specific retrieval counts, multistep deduplication, a source-level exclusion log, a literature-selection flow diagram, clinical publication-lineage mapping, and registry-status verification. However, the review did not use a standardized risk-of-bias instrument or meta-analysis, and full articles were not required for every nonclinical source used only for high-level qualitative context when an indexed abstract was sufficient to establish the relevant mechanism. Detailed numerical, causal, and study-level clinical statements were verified against complete primary sources whenever available. The synthesis should therefore be interpreted as structured and auditable rather than as a formal systematic review.

Four priorities emerge from the evidence: confirm platform-specific tumor exposure and productive infection or payload expression; resolve which liver-resident cells mediate clearance, antigen presentation, spatial exclusion, or toxicity; test incremental efficacy against contemporary matched control arms; and evaluate tumor control together with longitudinal hepatic reserve. Table 5 translates these priorities into program-level development considerations rather than patient-level treatment rules.

**TABLE 5:** Program-level post-PHOCUS development considerations. These considerations are intended for trial and platform development and should not be applied as patient-level treatment rules.

Development priority	Testable question	Priority evidence for prospective evaluation	Interpretation for program refinement
Platform-specific susceptibility and pharmacodynamics	Can selected HCC lesions be exposed and support productive infection or functional payload expression?	Baseline platform markers plus mandatory on-treatment tumor confirmation using prespecified platform-matched assays	Failure to demonstrate tumor exposure, productive infection, or functional payload expression suggests revisiting vector, route, dose, schedule, or assay strategy before efficacy expansion
Liver-resident and spatial mechanism	Which cells clear virus, present antigen, impose stromal exclusion, support antitumor immunity, or mediate liver toxicity?	Paired spatial tissue analysis, matched blood pharmacodynamics, lesion-level imaging, and tumor versus non-tumoral liver assessment	Predominantly non-tumor-directed, spatially ineffective, or hepatotoxic inflammation would support revising route, schedule, dose, or payload
Comparative efficacy and liver-safe benefit	Does the OV add benefit to a contemporary ICI or standardized locoregional backbone without compromising hepatic reserve?	Randomized comparator with the same non-OV backbone, prespecified liver-specific safety endpoints, decompensation adjudication, and subsequent-treatment access	Incremental benefit requires a matched comparator; deterioration in hepatic reserve can outweigh radiographic activity when judging net clinical value
Manufacturing and biosafety readiness	Can the product be delivered reproducibly with preserved infectivity, stable payload function, and acceptable exposure or shedding?	GMP release testing, genomic and infectious titers, mechanism-matched potency, route-specific handling stability, and platform-specific shedding assessment	Broader multicenter development is most interpretable after product performance, handling stability, and biosafety procedures are reproducible across sites

## 9 Conclusion

OV development in HCC is best considered a liver-centered spatial pharmacology problem involving susceptible malignant cells, liver-resident clearance and antigen-presenting compartments, fibrotic stroma, resident and recruited lymphocytes, non-tumoral diseased parenchyma, and anatomically distinct tumor sites. Current evidence supports feasibility and pharmacodynamic activity for selected platforms and defines a negative clinical boundary for Pexa-Vec followed by sorafenib, while the incremental efficacy of OV-containing regimens remains to be established in contemporary comparator-controlled studies.

Progress will depend on linking platform-specific tumor exposure and productive infection or functional payload expression to spatially resolved immune effects, clinically relevant tumor control, and preservation of hepatic reserve. VIRAL-HCC can serve as a non-scored organizational matrix for these measurements, but its value lies in structuring prospective hypotheses and biospecimens rather than directing treatment. Route- and schedule-specific, falsifiable trials are more likely to advance the field than class-wide assumptions or increasingly complex combinations without matched tumor and liver pharmacodynamics.
