# Supplementary material for: Liver-resident cellular determinants of oncolytic virotherapy in hepatocellular carcinoma: viral susceptibility and spatial immune remodeling
Source: Front Cell Dev Biol. 2026 Oct 7;14:1959317. doi: 10.3389/fcell.2026.1959317 (PMC13683942; doi:10.3389/fcell.2026.1959317)
Supplement: Supplementary file 1 [file DataSheet1.docx]

Supplementary Material 1

**Search strategy, screening audit, and evidence-appraisal procedures**

*Final bibliographic search: database inception to 5 September 2026 | Registry status reverified: 6 September 2026*

# 1. Search design and final audit

This structured critical narrative review used prespecified database-specific searches in PubMed/MEDLINE, Embase, and Web of Science Core Collection. The strategy combined a core HCC/oncolytic-virus concept with supplementary liver-directed delivery and liver-cellular/spatial-mechanism components. English-language limits were applied to bibliographic databases but not to trial registries. The three bibliographic searches were rerun for revision on 5 September 2026; trial-registry status and publication linkage were reverified on 6 September 2026.

Records were deduplicated using DOI, PMID, normalized title, first author, and publication year, followed by manual adjudication of ambiguous matches. Initial study identification and eligibility assessment were performed by DH; DH and QW independently verified design characteristics, numerical outcomes, correction status, registry status, and major safety findings of key clinical studies. Formal dual-independent screening of every retrieved record was not performed. The review was not conducted as a systematic review or meta-analysis; no formal risk-of-bias instrument, certainty framework, or quantitative pooling was applied.

**Supplementary Table S1. Final database retrieval and evidence-selection audit.**

| **Audit stage** | **n** | **Interpretation** |
| --- | --- | --- |
| PubMed/MEDLINE | 1,480 | Final revision export |
| Embase | 2,526 | Final revision export |
| Web of Science Core Collection | 2,106 | Final revision export |
| Total database records | 6,112 | Before deduplication |
| Duplicate records removed | 2,552 | DOI/PMID/title/author-year + manual adjudication |
| Unique records screened | 3,560 | Title/abstract audit |
| Excluded at title/abstract stage | 2,942 | Final screened-out count |
| Retained for source-level verification | 618 | 66 clinical; 305 HCC preclinical; 58 liver-directed; 189 mechanistic |
| Source-level exclusions | 23 | Report-level reasons in Supplementary Table S2 |
| Included reports in final evidence map | 595 | 65 clinical; 292 HCC preclinical; 57 liver-directed; 181 mechanistic |
| Unique HCC clinical study/evidence units | 26 | Derived from 65 HCC clinical reports after publication-lineage adjudication; nonclinical/cross-context evidence retained at report level |

Unit-of-analysis note: The 65 HCC clinical reports were consolidated into 26 unique clinical study/evidence units after publication-lineage adjudication. The 530 nonclinical/cross-context reports were retained and synthesized at report level and were not further consolidated into unique study units.

# 2. Complete bibliographic search strategies

*The final retrieval set in each database was the union of the core HCC search, the liver-directed delivery-context search, and the focused liver-cellular/spatial-mechanism search.*

**Search Table A1. PubMed/MEDLINE search strategy.**

| **Line** | **Search string** |
| --- | --- |
| 1 | "Carcinoma, Hepatocellular"[Mesh] OR hepatocellular carcinoma[tiab] OR HCC[tiab] OR primary liver cancer[tiab] OR liver cancer[tiab] |
| 2 | "Oncolytic Virotherapy"[Mesh] OR oncolytic virus*[tiab] OR oncolytic virotherap*[tiab] OR viroimmunotherap*[tiab] OR oncolytic immunotherap*[tiab] OR virus-mediated oncolysis[tiab] OR adenovirus[tiab] OR adenoviral[tiab] OR H101[tiab] OR rhAd5[tiab] OR dl1520[tiab] OR ONYX-015[tiab] OR vaccinia[tiab] OR JX-594[tiab] OR Pexa-Vec[tiab] OR pexastimogene devacirepvec[tiab] OR herpes simplex virus[tiab] OR HSV-1[tiab] OR VG161[tiab] OR talimogene laherparepvec[tiab] OR T-VEC[tiab] OR TG6002[tiab] OR reovirus[tiab] OR vesicular stomatitis virus[tiab] OR VSV[tiab] OR Newcastle disease virus[tiab] OR NDV[tiab] OR measles virus[tiab] OR coxsackievirus[tiab] |
| 3 | #1 AND #2 |
| 4 | "Liver Neoplasms"[Mesh] OR liver neoplasm*[tiab] OR liver tumor*[tiab] OR liver tumour*[tiab] OR hepatic tumor*[tiab] OR hepatic tumour*[tiab] OR liver metastas*[tiab] OR hepatic metastas*[tiab] |
| 5 | intratumor*[tiab] OR intralesional[tiab] OR hepatic arterial[tiab] OR intra-arterial[tiab] OR intraarterial[tiab] OR transarterial[tiab] OR intravenous[tiab] OR regional deliver*[tiab] OR liver-directed[tiab] |
| 6 | #2 AND #4 AND #5 |
| 7 | cirrho*[tiab] OR portal hypertension[tiab] OR hepatic reserve[tiab] OR Child-Pugh[tiab] OR ALBI[tiab] OR Kupffer[tiab] OR liver sinusoidal endothelial[tiab] OR dendritic cell*[tiab] OR antigen presentation[tiab] OR cross-presentation[tiab] OR hepatic stellate cell*[tiab] OR cancer-associated fibroblast*[tiab] OR extracellular matrix[tiab] OR macrophage*[tiab] OR myeloid[tiab] OR complement[tiab] OR neutralizing antibod*[tiab] OR neutralising antibod*[tiab] OR interferon[tiab] OR cGAS[tiab] OR STING[tiab] OR cGAS-STING[tiab] OR RIG-I[tiab] OR MDA5[tiab] OR Toll-like receptor*[tiab] OR immune exclusion[tiab] OR beta-catenin[tiab] OR β-catenin[tiab] OR single-cell[tiab] OR spatial transcriptom*[tiab] OR multiplex imaging[tiab] OR immune cell localization[tiab] |
| 8 | #1 AND #2 AND #7 |
| 9 | (#3 OR #6 OR #8) AND English[lang] |

**Search Table A2. Embase.com search strategy.**

| **Line** | **Search string** |
| --- | --- |
| 1 | 'hepatocellular carcinoma'/exp OR 'hepatocellular carcinoma':ti,ab,kw OR hcc:ti,ab,kw OR 'primary liver cancer':ti,ab,kw OR 'liver cancer':ti,ab,kw |
| 2 | 'oncolytic virus'/exp OR 'oncolytic virotherapy':ti,ab,kw OR 'oncolytic virus*':ti,ab,kw OR viroimmunotherap*:ti,ab,kw OR 'oncolytic immunotherap*':ti,ab,kw OR adenovirus:ti,ab,kw OR adenoviral:ti,ab,kw OR h101:ti,ab,kw OR rhad5:ti,ab,kw OR dl1520:ti,ab,kw OR 'onyx-015':ti,ab,kw OR vaccinia:ti,ab,kw OR 'jx-594':ti,ab,kw OR 'pexa-vec':ti,ab,kw OR 'pexastimogene devacirepvec':ti,ab,kw OR 'herpes simplex virus':ti,ab,kw OR 'hsv-1':ti,ab,kw OR vg161:ti,ab,kw OR 'talimogene laherparepvec':ti,ab,kw OR 't-vec':ti,ab,kw OR tg6002:ti,ab,kw OR reovirus:ti,ab,kw OR 'vesicular stomatitis virus':ti,ab,kw OR vsv:ti,ab,kw OR 'newcastle disease virus':ti,ab,kw OR ndv:ti,ab,kw OR 'measles virus':ti,ab,kw OR coxsackievirus:ti,ab,kw |
| 3 | #1 AND #2 |
| 4 | 'liver neoplasm*':ti,ab,kw OR 'liver tumor*':ti,ab,kw OR 'liver tumour*':ti,ab,kw OR 'hepatic tumor*':ti,ab,kw OR 'hepatic tumour*':ti,ab,kw OR 'liver metastas*':ti,ab,kw OR 'hepatic metastas*':ti,ab,kw |
| 5 | intratumor*:ti,ab,kw OR intralesional:ti,ab,kw OR 'hepatic arterial':ti,ab,kw OR 'intra-arterial':ti,ab,kw OR intraarterial:ti,ab,kw OR transarterial:ti,ab,kw OR intravenous:ti,ab,kw OR 'regional deliver*':ti,ab,kw OR 'liver-directed':ti,ab,kw |
| 6 | #2 AND #4 AND #5 |
| 7 | cirrho*:ti,ab,kw OR 'portal hypertension':ti,ab,kw OR 'hepatic reserve':ti,ab,kw OR 'child-pugh':ti,ab,kw OR albi:ti,ab,kw OR kupffer:ti,ab,kw OR 'liver sinusoidal endothelial':ti,ab,kw OR 'dendritic cell*':ti,ab,kw OR 'antigen presentation':ti,ab,kw OR 'cross-presentation':ti,ab,kw OR 'hepatic stellate cell*':ti,ab,kw OR 'cancer-associated fibroblast*':ti,ab,kw OR 'extracellular matrix':ti,ab,kw OR macrophage*:ti,ab,kw OR myeloid:ti,ab,kw OR complement:ti,ab,kw OR 'neutralizing antibod*':ti,ab,kw OR 'neutralising antibod*':ti,ab,kw OR interferon:ti,ab,kw OR cgas:ti,ab,kw OR sting:ti,ab,kw OR 'cgas-sting':ti,ab,kw OR 'rig-i':ti,ab,kw OR mda5:ti,ab,kw OR 'toll-like receptor*':ti,ab,kw OR 'immune exclusion':ti,ab,kw OR 'beta-catenin':ti,ab,kw OR 'single-cell':ti,ab,kw OR 'spatial transcriptom*':ti,ab,kw OR 'multiplex imaging':ti,ab,kw OR 'immune cell localization':ti,ab,kw |
| 8 | #1 AND #2 AND #7 |
| 9 | (#3 OR #6 OR #8) AND [english]/lim |

**Search Table A3. Web of Science Core Collection search strategy.**

| **Line** | **Search string** |
| --- | --- |
| 1 | TS=("hepatocellular carcinoma" OR HCC OR "primary liver cancer" OR "liver cancer") |
| 2 | TS=("oncolytic virus*" OR "oncolytic virotherap*" OR viroimmunotherap* OR "oncolytic immunotherap*" OR adenovirus OR adenoviral OR H101 OR rhAd5 OR dl1520 OR ONYX-015 OR vaccinia OR JX-594 OR Pexa-Vec OR "pexastimogene devacirepvec" OR "herpes simplex virus" OR HSV-1 OR VG161 OR "talimogene laherparepvec" OR T-VEC OR TG6002 OR reovirus OR "vesicular stomatitis virus" OR VSV OR "Newcastle disease virus" OR NDV OR "measles virus" OR coxsackievirus) |
| 3 | #1 AND #2 |
| 4 | TS=(("liver neoplasm*" OR "liver tumor*" OR "liver tumour*" OR "hepatic tumor*" OR "hepatic tumour*" OR "liver metastas*" OR "hepatic metastas*") AND (intratumor* OR intralesional OR "hepatic arterial" OR intra-arterial OR intraarterial OR transarterial OR intravenous OR "regional deliver*" OR "liver-directed")) |
| 5 | #2 AND #4 |
| 6 | TS=(cirrho* OR "portal hypertension" OR "hepatic reserve" OR Child-Pugh OR ALBI OR Kupffer OR "liver sinusoidal endothelial" OR "dendritic cell*" OR "antigen presentation" OR "cross-presentation" OR "hepatic stellate cell*" OR "cancer-associated fibroblast*" OR "extracellular matrix" OR macrophage* OR myeloid OR complement OR "neutralizing antibod*" OR "neutralising antibod*" OR interferon OR cGAS OR STING OR "cGAS-STING" OR "RIG-I" OR MDA5 OR "Toll-like receptor*" OR "immune exclusion" OR beta-catenin OR "single-cell" OR "spatial transcriptom*" OR "multiplex imaging" OR "immune cell localization") |
| 7 | #1 AND #2 AND #6 |
| 8 | #3 OR #5 OR #7 |
| 9 | Refine #8 by language: English |

# 3. Trial-registry search strategies

ClinicalTrials.gov searches were restricted to interventional studies without a recruitment-status restriction. WHO ICTRP searches were run as separate simple keyword combinations because indexing varies across source registries. Registry records were used to describe protocol characteristics and development activity, not efficacy.

**Search Table A4. ClinicalTrials.gov searches.**

| **Search** | **Condition or disease** | **Other terms** |
| --- | --- | --- |
| 1 | Hepatocellular carcinoma OR liver cancer | oncolytic virus |
| 2 | Hepatocellular carcinoma OR liver cancer | H101 |
| 3 | Hepatocellular carcinoma OR liver cancer | Pexa-Vec OR JX-594 OR pexastimogene devacirepvec |
| 4 | Hepatocellular carcinoma OR liver cancer | VG161 |
| 5 | Hepatocellular carcinoma OR liver cancer | dl1520 OR ONYX-015 |
| 6 | Hepatocellular carcinoma OR liver cancer | adenovirus OR vaccinia OR herpes simplex virus |

**Search Table A5. WHO ICTRP searches.**

| **Search** | **Keyword combination** |
| --- | --- |
| 1 | hepatocellular carcinoma AND oncolytic virus |
| 2 | liver cancer AND oncolytic virus |
| 3 | hepatocellular carcinoma AND H101 |
| 4 | hepatocellular carcinoma AND Pexa-Vec |
| 5 | hepatocellular carcinoma AND JX-594 |
| 6 | hepatocellular carcinoma AND VG161 |
| 7 | hepatocellular carcinoma AND adenovirus |
| 8 | hepatocellular carcinoma AND vaccinia |
| 9 | hepatocellular carcinoma AND herpes simplex virus |

# 4. Eligibility, source handling, and evidence annotation

Eligible evidence comprised original HCC clinical studies of oncolytic viruses; HCC-specific in vitro or in vivo studies addressing viral susceptibility, immune remodeling, delivery, combination strategies, or liver-specific toxicity; non-HCC liver-directed OV studies when directly informative for route-specific development; and non-HCC mechanistic studies when directly relevant to a prespecified HCC hypothesis. Public registry records and meeting abstracts were used for development status, dose selection, or preliminary pharmacodynamic context rather than comparative efficacy.

Excluded evidence comprised non-oncolytic viral gene therapy or vaccine studies without a replicating OV component, non-HCC studies without direct mechanistic/delivery/safety relevance, duplicate or superseded reports without distinct information, retracted publications, and unverifiable unpublished claims. Peer-reviewed full articles were preferred for numerical or causal statements. Complete meeting abstracts were treated as the complete available source when no stand-alone article existed. Corrections/errata were linked to the primary article; retracted reports were excluded.

**Supplementary Table S3. Three-dimensional evidence-annotation framework.**

| **Dimension** | **Categories** | **Interpretive purpose** |
| --- | --- | --- |
| Study design | Randomized clinical trial; prospective non-randomized/early-phase study; retrospective/observational study; case report/series; preclinical experimental study | Describes methodological design without conflating it with disease context. |
| Biological context | HCC clinical; mixed liver cancer including HCC; HCC preclinical; non-HCC liver-directed; other-cancer mechanistic | Specifies where the evidence was generated. |
| Permissible inference | Comparative efficacy; feasibility/safety; viral pharmacodynamics; immune pharmacodynamics; biological mechanism; hypothesis generation | Defines the strongest conclusion supported by that source. |
| Publication-status qualifier | Peer-reviewed full article; meeting abstract; registry record; correction/erratum; retraction notice | Qualifies source maturity without creating a pseudo-numeric evidence hierarchy. |

# 5. Literature identification and source-level audit

Supplementary Figure S1. Literature identification and source-level evidence audit. HCC-clinical reports were consolidated into 26 unique clinical study/evidence units; nonclinical and cross-context evidence was retained at report level. The diagram documents audit transparency for a structured critical narrative review and does not imply a formal systematic-review design.

# 6. Source-level/full-text exclusions

*Codes: FT2, insufficient direct HCC/liver relevance; FT4, conference/secondary report without sufficient distinct mature primary evidence for the final map; FT5, duplicate/overlapping/superseded report without additional relevant information. No excluded report was used to support a quantitative clinical conclusion.*

**Supplementary Table S2. Source-level/full-text exclusions and reasons.**

| **Record ID** | **Year / context** | **Title** | **Code** | **Specific reason** |
| --- | --- | --- | --- | --- |
| OVD1857 | 2013 \| HCC clinical | Randomized dose-finding clinical trial of oncolytic immunotherapeutic vaccinia JX-594 in liver cancer | FT5 | Citation/database artifact with the same PMID/DOI as the primary Nature Medicine report OVD1856; no distinct evidentiary content. |
| OVD0183 | 2025 \| HCC preclinical | Systemic delivery of glycosylated-PEG-masked oncolytic virus enhances targeting of antitumor immuno-virotherapy and modulates T and NK cell infiltration | FT4 | Conference-only nonclinical report; insufficient distinct mature source for the final mechanism evidence map. |
| OVD0605 | 2022 \| HCC preclinical | Therapeutic efficacy of an oncolytic influenza virus carrying an antibody against programmed cell death 1 in hepatocellular carcinoma | FT4 | Conference-only nonclinical report; not retained as a stand-alone mechanistic evidence unit. |
| OVD1596 | 2014 \| HCC preclinical | Combined anti-viral and anti-tumour therapy for virus-associated liver cancer | FT4 | Conference-only report; insufficient distinct detail for the final evidence map. |
| OVD1662 | 2014 \| HCC preclinical | PET imaging of oncolytic VSV expressing the mutant HSV-1 thymidine kinase transgene in a preclinical HCC rat model | FT4 | Conference-only report; not retained as a stand-alone evidence unit. |
| OVD1698 | 2014 \| HCC preclinical | The effect of a targeted poxvirus (JX-594) on Hepatocellular Carcinoma (HCC) derived cell lines | FT4 | Conference-only report; not retained as a stand-alone evidence unit. |
| OVD1743 | 2013 \| HCC preclinical | Bioreducible polymer-conjugated oncolytic adenovirus for hepatoma-specific therapy via systemic administration | FT4 | Conference-only report; not retained as a stand-alone evidence unit. |
| OVD2084 | 2011 \| HCC preclinical | Antifibrotic properties of transarterial VSV oncolytic therapy for hepatocellular carcinoma in rats with thioacetamide-induced liver fibrosis | FT5 | Conference report overlapping with the same program/title represented elsewhere; no distinct mature source retained. |
| OVD2085 | 2011 \| HCC preclinical | Antifibrotic properties of transarterial VSV oncolytic therapy for hepatocellular carcinoma in rats with thioacetamide-induced liver fibrosis | FT5 | Duplicate/overlapping conference report of the same preclinical program; no distinct evidentiary contribution. |
| OVD2129 | 2011 \| HCC preclinical | Enhanced oncolytic efficacy after polymer coating of an α-fetoprotein promoter driven, conditionally replicating adenovirus that expresses the sodium iodide symporter (NIS) | FT4 | Conference-only report; not retained as a stand-alone evidence unit. |
| OVD2173 | 2011 \| HCC preclinical | Oncolytic adenoviral vector targeted to hepatocellular carcinoma expressing low levels of miR-122 | FT4 | Conference-only report; insufficient mature primary detail for final synthesis. |
| OVD2174 | 2011 \| HCC preclinical | Oncolytic adenovirus regulated by modified AFP promoter induces hepatocellular carcinoma-specific and hypoxia-independent cell killing | FT4 | Conference-only report; insufficient mature primary detail for final synthesis. |
| OVD2188 | 2011 \| HCC preclinical | Radioiodine imaging-guided analysis of therapeutic efficacy of an α-fetoprotein promoter driven, conditionally replicating adenovirus that expresses the sodium iodide symporter (NIS) | FT4 | Conference-only report; not retained as a stand-alone evidence unit. |
| OVD2457 | 2009 \| HCC preclinical | Proteasome inhibition during oncolytic infection improves antitumoral immunity and inhibits metastatic tumor growth | FT4 | Conference-only report; not retained as a stand-alone evidence unit. |
| OVD1129 | 2018 \| Liver-directed cross-context | The efficacy of oncolytic virus therapy using G47Δ in mouse biliary tract cancer models | FT2 | Non-HCC preclinical study without sufficient direct route-specific delivery or liver-safety relevance to the questions retained in this review. |
| OVD1171 | 2017 \| Mechanistic cross-context | Enhancing the Oncolytic Activity of CD133-Targeted Measles Virus: Receptor Extension or Chimerism with Vesicular Stomatitis Virus Are Most Effective | FT2 | Cross-context engineering study not directly required for the prespecified HCC/liver mechanism claims retained in the final review. |
| OVD1354 | 2016 \| Mechanistic cross-context | ASGCT 19th Annual Meeting: Abstracts | FT5 | Meeting-abstract compendium/database record rather than a distinct report; duplicated conference-container metadata. |
| OVD1370 | 2016 \| Mechanistic cross-context | ASGCT 19th Annual Meeting: Abstracts | FT5 | Meeting-abstract compendium/database record rather than a distinct report; duplicated conference-container metadata. |
| OVD1679 | 2014 \| Mechanistic cross-context | Role of apoptotic signaling in susceptibility of pancreatic cancer cells to vesicular stomatitis virus-mediated oncolysis | FT2 | Pancreatic-cancer mechanism not sufficiently direct for the liver/HCC mechanistic questions retained in the final synthesis. |
| OVD1797 | 2013 \| Mechanistic cross-context | In vivo expansion of a recipient population of cell carriers allows for highly effective systemic delivery of oncolytic reovirus even in the presence of neutralizing antibody | FT4 | Conference-only cross-context report; not retained as a stand-alone final evidence unit. |
| OVD2027 | 2012 \| Mechanistic cross-context | Selective targeting and disruption of tumor-associated vasculature in humans with an engineered oncolytic poxvirus | FT5 | Conference report superseded by a peer-reviewed mature publication on the same vascular mechanism. |
| OVD2102 | 2011 \| Mechanistic cross-context | Clinical trial results: Intralesional injection of a tumor selective oncolytic vaccinia virus | FT5 | Conference report superseded/overlapped by mature peer-reviewed clinical publications. |
| OVD2113 | 2011 \| Mechanistic cross-context | Demonstration of safety and antitumoral activity of JX-594, a targeted multi-mechanistic oncolytic poxvirus, following intravenous infusion and/or intratumoral injections in patients with refractory metastatic cancers | FT5 | Conference report superseded/overlapped by mature peer-reviewed clinical publications. |

# 7. HCC clinical study/evidence-unit inventory

The 65 retained HCC-clinical reports were conservatively consolidated into 26 unique clinical study/evidence units. Multiple reports from the same program were linked by trial identifier, platform, population, and publication lineage. The 2012–2013 H101+TACE report was conservatively merged into the larger 2007–2015 same-center Sun Yat-sen cohort family. A pooled/multi-trial JX-594 conference lineage was not counted as a unique study.

**Supplementary Table S4. Final HCC clinical study/evidence-unit inventory.**

| **Unit** | **Study/program** | **Primary source** | **Registry** | **Design** | **Permissible inference** | **Lineage** |
| --- | --- | --- | --- | --- | --- | --- |
| C01 | RP2/RP3 HCC development program | OVD0003 DOI: 10.1200/jco.2026.44.2_suppl.tps614 | NCT05733598 | Phase 2 protocol/meeting-abstract development | Development status and feasibility only; no efficacy inference. | 6 linked report(s); registry/program linkage remains source-limited where noted |
| C02 | Pexa-Vec + nivolumab long-term case/transplant follow-up | OVD0035 DOI: 10.1007/s12029-026-01573-0 | Registry ID unresolved | Patient-level case report from a phase I/IIa program | Individual downstaging/transplant observation only. | 2 linked report(s); registry/program linkage remains source-limited where noted |
| C03 | M1-c6v1 + camrelizumab + apatinib | OVD0097 DOI: 10.1158/1078-0432.ccr-25-1620 | NCT04665362 | Single-arm investigator-initiated clinical study | Safety/feasibility/PD and preliminary activity; no comparative efficacy. | 2 linked report(s) |
| C04 | H101 + tislelizumab abscopal-effect case | OVD0139 DOI: 10.62347/vsqy6496 | Not registered in supplied source | Case report | Spatial/immune hypothesis generation only. | 1 linked report(s) |
| C05 | VG161 + camrelizumab | OVD0155 DOI: 10.1200/jco.2025.43.16_suppl.e14605 | NCT06124001 | Phase Ib/IIa abstract-level study | Development/feasibility signal only. | 1 linked report(s) |
| C06 | VG161 monotherapy | OVD0156 DOI: 10.1038/s41586-025-08717-5 | NCT04806464 | Multicenter phase I | Feasibility and pharmacodynamics; exploratory biomarker findings; no comparative efficacy. | 3 linked report(s) |
| C07 | MASTERKEY-318: intrahepatic T-VEC ± pembrolizumab | OVD0170 DOI: 10.1093/oncolo/oyae203 | NCT02509507 | Phase Ib/II multicenter open-label basket trial | Route-specific safety/feasibility and HCC cohort activity; non-comparative. | 7 linked report(s) |
| C08 | H101 + RFA randomized trial | OVD0176 DOI: 10.1097/js9.0000000000004438 | NCT03790059 | Prospective randomized phase III | Comparative evidence for the tested regimen; no significant DFS/OS improvement. | 1 linked report(s) |
| C09 | H101 + TAE + sequential thermal ablation retrospective cohort | OVD0190 DOI: 10.3389/fonc.2025.1623877 | No registry ID identified | Single-center retrospective cohort | Feasibility/outcome description; no causal additive-effect inference. | 1 linked report(s) |
| C10 | H101 + TAE + sequential thermal ablation prospective study | OVD0341 DOI: 10.1016/j.annonc.2023.09.2971 | No registry ID identified | Prospective randomized trial-in-progress abstract | Study design/development only until mature results are available. | 1 linked report(s) |
| C11 | Multimodal intra-arterial/local OV case | OVD0232 DOI: 10.2174/1568009623666230801093031 | No registry ID identified | Case report + literature review | Hypothesis-generating multimodal observation only. | 1 linked report(s) |
| C12 | H101 + nivolumab pilot | OVD0325 DOI: 10.1016/j.esmoop.2024.102239 | ChiCTR1900025377 | Open-label single-arm pilot | Feasibility/safety and immune-priming hypothesis; no additive-efficacy attribution. | 2 linked report(s); HCC pilot registered as ChiCTR1900025377; erroneous NCT cross-link removed |
| C13 | PHOCUS | OVD0426 DOI: 10.1159/000533650 | NCT02562755 | Randomized open-label phase III | Negative comparative efficacy for the tested Pexa-Vec→sorafenib sequence; not class-wide OV futility. | 2 linked report(s) |
| C14 | OBP-301/suratadenoturev phase I | OVD0450 DOI: 10.1016/j.ymthe.2023.04.006 | NCT02293850 | Phase I open-label dose escalation | Safety/dose/local PD; no comparative efficacy. | 3 linked report(s) |
| C15 | Postoperative rhAd5/H101 + TACE cohort | OVD0656 DOI: 10.21037/jgo-21-792 | No registry ID identified | Observational postoperative cohort | Association with recurrence/metastasis outcomes; viral contribution not isolated. | 1 linked report(s) |
| C16 | JX-594/Pexa-Vec + sorafenib patient-level case | OVD0783 DOI: 10.17998/jlc.20.2.177 | No registry ID identified | Case report | Individual observation only. | 1 linked report(s) |
| C17 | Voyager-V1 + cemiplimab development study | OVD0834 DOI: 10.1200/jco.2020.38.15_suppl.tps3161 | Registry ID not resolved in supplied package | Phase II multi-tumor meeting abstract | Development/biomarker rationale only. | 1 linked report(s); registry/program linkage remains source-limited where noted |
| C18 | TRAVERSE | OVD0990 DOI: 10.1080/2162402x.2019.1615817 | NCT01387555 | Randomized open-label phase IIb | Randomized negative evidence in the post-sorafenib setting. | 4 linked report(s) |
| C19 | H101 + TACE overlapping Sun Yat-sen cohort family (2007–2015) | OVD1261 DOI: 10.1186/s40880-017-0227-2 | No registry ID identified | Retrospective comparative/PSM + secondary prognostic analyses | Observational association only; overlapping reports counted once. | 4 linked report(s); overlapping same-center reports counted once |
| C20 | JX-594/Pexa-Vec randomized dose-finding phase II | OVD1856 DOI: 10.1038/nm.3089 | NCT00554372 | Randomized dose-finding phase II | Biological proof-of-mechanism/dose-related signal; not active-comparator efficacy. | 5 linked report(s) |
| C21 | Pexa-Vec/JX-594 followed by sorafenib phase II program | OVD2197 DOI: 10.1038/mt.2011.39 | NCT01171651 | Single-arm phase II/pilot/PD program | Feasibility/PD and single-arm activity; cannot establish superiority or resensitization. | 6 linked report(s) |
| C22 | JX-594 phase I liver-tumor program | OVD2591 DOI: 10.1016/s1470-2045(08)70107-4 | NCT00629759 | Phase I dose escalation / HCC subreports | Early safety, replication, dissemination and pharmacodynamic proof-of-mechanism. | 2 linked report(s) |
| C23 | rhAd5/H101 + TACE controlled cohort | OVD1622 DOI: 10.1007/s12032-014-0095-4 | No registry ID identified | Retrospective controlled clinical study | Controlled association; no randomized causal inference. | 1 linked report(s) |
| C24 | H101 + TACE recurrent-HCC case | OVD2210 DOI: 10.3748/wjg.v17.i18.2353 | No registry ID identified | Case report | Individual feasibility observation only. | 1 linked report(s) |
| C25 | dl1520/ONYX-015 randomized prospective study | OVD3077 DOI: 10.1038/sj.cgt.7700431 | No registry ID identified | Small randomized prospective study vs ethanol | Early feasibility/safety and preliminary response; underpowered efficacy. | 2 linked report(s) |
| C26 | RFA + oncolytic adenovirus + anti-PD-1 two-case series | OVD0626 DOI: 10.1177/03000605211044596 | No registry ID identified | Two-case report | Hypothesis-generating multimodal observation only. | 1 linked report(s) |

# 8. Trial-registry status re-verification

Registry status was rechecked on 6 September 2026 and is used only to describe development activity. “Unknown/stale” is retained when a public record is not current rather than inferring completion from publication alone.

**Supplementary Table S5. Trial-registry status for HCC OV development programs.**

| **Registry ID** | **Program** | **Status** | **Enrollment** | **Phase** | **Publication/status note** |
| --- | --- | --- | --- | --- | --- |
| NCT00629759 | JX-594/Pexa-Vec phase I liver-tumor study | Completed | 14 | Phase 1 | Updated 19 May 2026; no registry results; primary peer-reviewed phase I publication linked. |
| NCT01387555 | TRAVERSE | Completed | 129 | Phase 2 | Updated 11 Mar 2015; peer-reviewed TRAVERSE publication linked. |
| NCT02293850 | OBP-301/suratadenoturev phase I | Unknown status | 18 target | Phase 1 | Public registry record is stale (last verified 2018); 2023 peer-reviewed phase I report available. |
| NCT02509507 | MASTERKEY-318 / intrahepatic T-VEC ± pembrolizumab | Completed | 127 | Phase 1/2 | Updated 6 Aug 2024; results posted and 2025 peer-reviewed report linked. |
| NCT02562755 | PHOCUS | Completed | 459 | Phase 3 | Updated 8 Jun 2026; results posted and 2024 peer-reviewed report linked. |
| NCT03790059 | H101 + RFA randomized study | Unknown/stale registry status | 160 target; 162 in final publication | Not applicable / randomized trial | Registry record is stale; 2026 peer-reviewed randomized phase III report documents trial completion and 162 randomized patients. |
| NCT04665362 | M1-c6v1 + camrelizumab + apatinib | Unknown status | 10 target | Phase 1 | Updated 22 Jan 2021; last known status not yet recruiting; 2025 peer-reviewed clinical report now available. |
| NCT04806464 | VG161 monotherapy phase I | Recruiting | 44 | Phase 1 | Updated 19 Sep 2024; no results posted on registry; 2025 peer-reviewed publication available. |
| NCT05733598 | RP2 HCC development program | Recruiting | 60 | Phase 2 | Updated 2 Apr 2026; no results posted; current record describes RP2 study. |
| NCT06124001 | VG161 + camrelizumab | Unknown status | 36 target | Phase 1/2 | Updated 15 Nov 2023; last known status not yet recruiting; 2025 conference abstract available. |
| NCT03071094 | Pexa-Vec + nivolumab | Terminated | 14 | Phase 1/2 | Terminated; results have been reported. Used only to describe development activity. |
| NCT05223816 | VG161 monotherapy/combination with nivolumab | Recruiting | 97 | Phase 2 | Updated 9 Aug 2024; no results posted on registry at verification. |
| ChiCTR1900025377 | H101 + nivolumab pilot | Completed publication available | 18 assessable | Pilot | Registration identifier reported in the peer-reviewed ESMO Open article; this replaces an erroneous NCT cross-link used in an earlier working audit. |

# 9. Nonclinical and cross-context evidence-map composition

**Supplementary Table S6. Final nonclinical and cross-context evidence-map composition.**

| **Evidence context** | **Retained reports** | **Primary use** | **Inference boundary** |
| --- | --- | --- | --- |
| HCC preclinical | 292 | Platform susceptibility, replication, payload, immune mechanism, route and diseased-liver safety | Supports HCC-specific mechanism; does not establish clinical efficacy. |
| Liver-directed cross-context | 57 | Hepatic/intratumoral delivery, biodistribution, hepatic clearance and route-specific safety | Used only when the liver-delivery question is explicit; not HCC efficacy evidence. |
| Mechanistic cross-context | 181 | Innate sensing, antiviral immunity, antigen presentation, spatial/immune remodeling and scheduling | Supports mechanistic plausibility; extrapolation to HCC is explicitly bounded. |

# 10. Final source-audit notes

Retracted publications were excluded from evidence synthesis. Corrections/errata were linked to their primary articles rather than counted independently; the VG161 Nature publisher correction was retained with the primary report. Shared conference-supplement DOIs were not used as stand-alone duplicate identifiers because multiple distinct abstracts can share a container DOI.

For H101 plus nivolumab, the final peer-reviewed publication identifies ChiCTR1900025377. An erroneous NCT cross-link used in an earlier working audit was removed from the HCC registry map because it corresponded to a pancreatic-cancer study rather than the HCC pilot. This correction prevents erroneous trial-lineage assignment.

Final evidence-map composition: 595 included reports, comprising 65 HCC clinical reports, 292 HCC preclinical reports, 57 liver-directed cross-context reports, and 181 mechanistic cross-context reports. After conservative publication-lineage adjudication, the 65 HCC clinical reports represented 26 unique clinical study/evidence units. The 530 nonclinical/cross-context reports were retained and synthesized at report level and were not further consolidated into unique study units.
